# Adolescent Binge Alcohol Enhances Early Alzheimer’s Disease Pathology in Adulthood Through Proinflammatory Neuroimmune Activation

**DOI:** 10.3389/fphar.2022.884170

**Published:** 2022-04-26

**Authors:** Alexandra Barnett, Emeraghi David, Aaron Rohlman, Viktoriya D. Nikolova, Sheryl S. Moy, Ryan P. Vetreno, Leon G. Coleman

**Affiliations:** ^1^ Department of Pharmacology, University of North Carolina at Chapel Hill School of Medicine, Chapel Hill, NC, United States; ^2^ Bowles Center for Alcohol Studies, University of North Carolina at Chapel Hill School of Medicine, Chapel Hill, NC, United States; ^3^ Department of Psychiatry, University of North Carolina at Chapel Hill School of Medicine, Chapel Hill, NC, United States; ^4^ Carolina Institute for Developmental Disorders, University of North Carolina at Chapel Hill School of Medicine, Chapel Hill, NC, United States

**Keywords:** neuroinflammation, Alzheiemer’s disease, adolescence, alcohol, amyloid, addiction

## Abstract

Epidemiological studies suggest that heavy alcohol use early in life is associated with increased risk for Alzheimer’s disease (AD). However, mechanisms connecting AD with alcohol use have not been identified. Both heavy alcohol use and AD feature increased proinflammatory signaling. Therefore, we hypothesized that adolescent binge ethanol would increase AD molecular and behavioral pathology in adulthood through proinflammatory signaling. The 3xTg-AD mouse model (APPSwe, tauP301, Psen1^tm1Mpm^) which features amyloid (Aβ) and tau pathology beginning at 6–12 months underwent adolescent intermittent ethanol (AIE, 5 g/kg/d, i.g., P25-55) with assessment of AD pathologic mediators at P200. A second group of mice received AIE +/− minocycline (30 mg/kg/d, IP) followed by behavioral testing in adulthood. Behavioral testing and age of testing included: locomotor activity and exploration (27–28 weeks), novel object recognition (NORT, 28-30 weeks), 3-chamber sociability and social memory (29–31 weeks), prepulse inhibition (PPI, 30–32 weeks), Morris Water Maze with reversal (MWM, 31–35 weeks), and Piezo sleep monitoring (35–37 weeks). We found that AIE increased levels of neurotoxic Aβ_1–42_ in adult female hippocampus as well as intraneuronal Aβ_1–42_ in amygdala and entorhinal cortex. Phosphorylated tau at residue Thr181 (p-tau-181) was also increased in female hippocampus by AIE. Several proinflammatory genes were persistently increased by AIE in the female hippocampus, including IL-1β, MCP-1, IL-6, and IFNα. Expression of these genes was strongly correlated with the levels of Aβ_1–42_ and p-tau-181 in hippocampus. AIE caused persistent decreases in locomotor activity (open-field and NORT habituation) and increased anxiety-like behavior (thigmotaxis) while reducing memory retention. Treatment with the anti-inflammatory compound minocycline during AIE blocked persistent increases in Aβ_1–42_ in amygdala and p-tau-181 in hippocampus, and prevented AIE-induced thigmotaxis and memory loss. Together, these data find that adolescent binge ethanol enhances AD molecular and behavioral pathology in adulthood through proinflammatory signaling. Blockade of proinflammatory signaling during ethanol exposure prevents ethanol-induced effects on pathologic accumulation of AD-associated proteins and persistent behavior changes relevant to human AD.

## 1 Introduction

Adolescence is a critical period in neurodevelopment, wherein the lasting structure of significant neurocircuitry and cognitive function is established (Lees et al., 2020). Therefore, environmental insults during adolescence can have long-lasting effects. Alcohol (i.e., ethanol) is the most commonly abused substance in adolescence, and can result in persistent deficits in learning, memory, cognitive function, and anxiety-like behavior (Squeglia et al., 2014; Spear, 2018). This unique ability of adolescent ethanol to negatively impact the adult neuro-environment raises the possibility that adolescent ethanol could increase the risk for neuropsychiatric disorders later in life. In fact, adolescent binge ethanol promotes progressive neurodegeneration of adult cholinergic neurons that are lost in Alzheimer’s disease (AD) (Coleman et al., 2011; Crews et al., 2019). Epidemiological studies support that heavy ethanol use increases risk for Alzheimer’s disease (AD) (Mukamal et al., 2003; Koch et al., 2019) with additional work finding that heavy use earlier in life increases risk for AD in late adulthood (Langballe et al., 2015). Further, heavy alcohol use was the number one modifiable risk factor for AD and all-cause dementia in a French study that assessed over a million patients (Schwarzinger et al., 2018). A recent preclinical study similarly found that adolescent binge ethanol increased AD pathology in an amyloid-expressing transgenic mouse model (Ledesma et al., 2021). Thus, binge alcohol use during adolescence is emerging as a key risk factor for AD.

AD is a progressive neurodegenerative disease that usually (>90%) manifests in late life (over the age of 65) (Hersi et al., 2017). The vast majority of AD cases have no identifiable etiology, though many risk factors have been identified such as female sex, heavy alcohol use, obesity, smoking, and diabetes (Schwarzinger et al., 2018). AD is diagnosed pathologically by the presence of both amyloid (Aβ) plaques and tau fibrils; however, the pathogenesis of AD remains unclear. Both AD and binge ethanol share commonalities such as neurodegeneration and induction of neuroimmune signaling (Zhang et al., 2013; Heneka et al., 2015; Crews et al., 2017; Efthymiou and Goate, 2017; Dani et al., 2018; Jack et al., 2018; Kinney et al., 2018). In both settings microglia are thought to contribute to pathology through the production of proinflammatory cytokines such as TNFα, IL-6, and IL-1β that can disrupt synaptic plasticity and promote neurodegeneration (Hansen et al., 2018). Neuroimmune gene induction is thought to promote the persistent neuronal deficits caused by adolescent binge ethanol (Coleman et al., 2021). Adolescence may represent a critical period for microglial function through microglial ”priming”. Binge ethanol during adolescence promotes priming of microglia to result in increased adult microglial responses to both ethanol and non-ethanol related stressors (Walter and Crews, 2017; Chastain et al., 2019). In AD, multiple studies report that pro-inflammatory microglial priming during vulnerable periods of life can enhance cognitive dysfunction and accelerate pathology (Hoeijmakers et al., 2016; Li et al., 2018). Thus, adolescent binge ethanol could potentially promote AD pathology through pro-inflammatory priming of microglia.

In this study we investigated the impact of adolescent binge ethanol on AD pathology using the 3xTg-AD mouse model (APPSwe, tauP301, Psen1^tm1Mpm^). The 3xTg-AD mouse model reproduces key features of human AD such as progressive accumulation of amyloid and tau with age-dependent cognitive decline (Oddo et al., 2003a; Belfiore et al., 2019). Aβ and tau pathology develop progressively with age and in a region-specific manner similar to findings in human AD (Billings et al., 2005). This model also features increased pathology in females (Carroll et al., 2010), similar to the sexual dimorphism seen in humans. Regions that are impacted early include the hippocampus, entorhinal cortex (ENT Cx) and amygdala (AMG) (Billings et al., 2005; Long and Holtzman, 2019). The accumulation of intraneuronal Aβ is an early pathologic lesion associated with early impairments in synaptic transmission and cognitive decline (Oddo et al., 2003b; Billings et al., 2005; Gouras et al., 2010). Development of neurofibrillary hyperphosphorylated and conformationally altered tau tangles is another hallmark of AD. Intraneuronal tau pathology first appears in 3xTg mice around 6 months (P180) of age in the pyramidal neurons of the hippocampus with later spreads to cortical regions (Belfiore et al., 2019), which mimics the distribution of tau in human AD brain (Billings et al., 2005). Tau is hyperphosphorylated at multiple residues leading to the formation of aggregates in human AD and 3xTg mice in an age and regional-dependent manner (Li and Gotz, 2017). This known temporal and brain-region associated pathologic spread in the 3xTg-AD mouse was used to determine the ages as which mice were assessed for early AD pathology.

Adolescents commonly abuse ethanol in an episodic binge drinking manner. Therefore, mice underwent adolescent intermittent ethanol (AIE) treatment paradigm that models the intermittent binge-drinking pattern of human adolescents (Crews et al., 2019). To determine if AIE promotes AD pathogenesis, mice were assessed in adulthood at an age with marked intraneuronal Aβ – a critical early lesion in AD (Billings et al., 2005; Gouras et al., 2010). We assessed the hippocampus, entorhinal cortex, and amygdala, regions impacted early in AD, for pathology and proinflammatory markers. We hypothesized that AIE would increase microglial proinflammatory signaling and worsen early AD pathology. We further hypothesized that inhibition of proinflammatory signaling during AIE would blunt AD molecular and behavioral pathology.

## 2 Materials and Methods

### 2.1 Animals

3xTg-AD breeders containing the human APPSwe, tauP301, and Psen1^tm1Mpm^ mutations were obtained from Jackson Labs through the Mutant Mouse Resource and Research Centers (MMRC). Homozygous breeder pairs were used, and pups were weaned on postnatal day 25 (P25). Animals were group-housed (up to 5/cage) with same-sex littermates in standard cages in a temperature and humidity-controlled vivarium on a 12 h/12 h light/dark cycle and had access to food and water as needed. All animal protocols were approved by the Institutional Animal Care and Use Committee (IACUC) at the University of North Carolina at Chapel Hill and were in accordance with NIH regulations (Protocols 20-232.0 and 21-052.0).

#### 2.1.1 Treatment and Timeline

**FIGURE 1:**
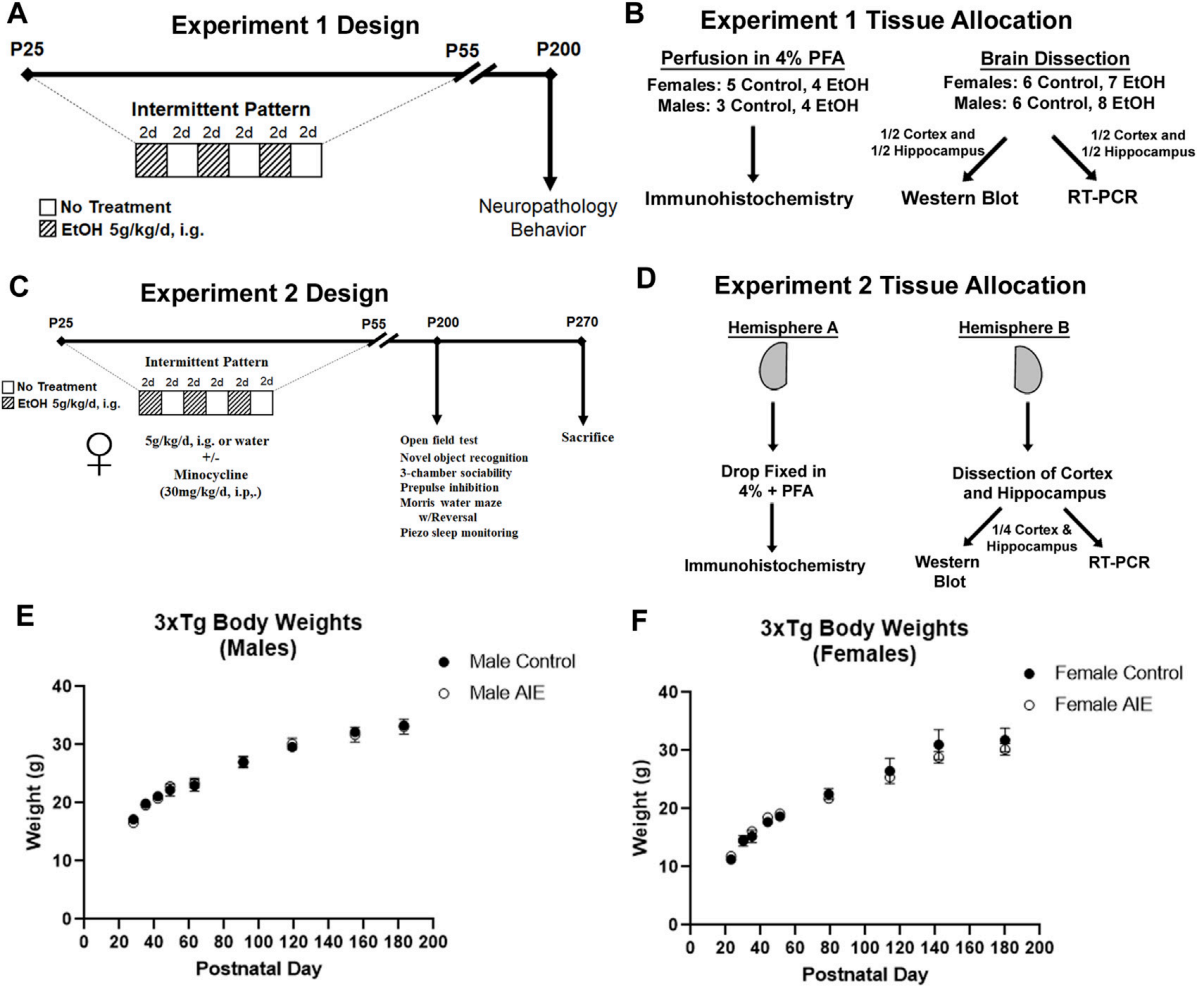
Experimental design and tissue allocation for both experiments. **(A, B)** Experiment 1. **(A)** Adolescent 3xTg male and female mice received either water gavage (i.g.) or AIE (5 g/kg/d, i.g.) from P25-55, in a 2-days on 2 days off pattern. Mice were then left without intervention until P200. **(B)** 16 mice were sacrificed by cardiac perfusion with 4% PFA for immunohistochemistry (Females: *N* = 5 control, 4 ethanol; Males: *N* = 3 control, 4 ethanol), with remaining mice perfused with PBS (Females: *N* = 6 control, 8 ethanol; Males: *N* = 6 control, 8 ethanol) and the cortex and hippocampus dissected and frozen in liquid nitrogen. One half of the cortex and hippocampus were prepared for western blot, while the other halves were prepared for RT-PCR. **(C, D)** Experiment 2. **(C)** Female mice received either water gavage + vehicle (i.p.) (*N* = 5), minocycline (Min, 30 mg/kg, i.p., *N* = 5), AIE (*N* = 9), or AIE + Min (*N* = 8) in the same schedule as Experiment 1, with behavioral testing beginning at P200. Mice were sacrificed at the end of the behavioral testing on P270. **(D)** Mice were sacrificed by cardiac perfusion with PBS and one hemisphere was drop-fixed in PFA for IHC and the other hemisphere dissected for cortex and hippocampus. Dissected cortex and hippocampus from one hemisphere were divided equally for western blot and RT-PCR analyses. **(E)** Body weights of male subjects across treatment and maturation into adulthood. **(F)** Body weights of female subjects across treatment and maturation into adulthood.

Adolescent male and female 3xTg-AD mice were randomly assigned to either water control or AIE treatment groups (Figures 1A,C). Mice received either ethanol (5 g/kg/d – 25% EtOH, w/v) or an equal volume of water per weight by intragastric gavage (i.g.) in a 2-day on/2-day off schedule from P25 to P55 as we have reported previously (Vetreno and Crews, 2012; Vetreno and Crews, 2018; Crews et al., 2019; Macht et al., 2020; Crews et al., 2021). This resulted in a total of 16 ethanol or water administrations. We have reported previously that this dose of ethanol results in a binge-level of intoxication with peak blood alcohol concentrations (BACs) at 1 h of 280–300 mg/dl (Coleman et al., 2011; Coleman et al., 2014). This high BAC is similar to blood levels reached by binge-drinking human adolescents who have been reported to intake ∼13 drinks/episode consistent with BACs of 250–300 (Deas et al., 2000; Jones and Holmgren, 2009). Mice were then left unperturbed from P55 to P200. Two cohorts of mice were treated. For cohort 1, 44 adolescent mice were separated into treatment groups: males (*N* = 9 control, *N* = 12 AIE) and females (*N* = 11 control, *N* = 12 AIE). Mice were sacrificed in adulthood (P200) by perfusion for immunohistochemistry (IHC) or tissue was frozen for RT-PCR and Western Blot. Brains from nine females (*N* = 5 control, 4 ethanol) and seven males (3 controls, 4 ethanol) were used for IHC. Brains from 15 females (*N* = 7 control, 8 ethanol) and 14 males (*N* = 6 control, 8 ethanol) were used for RT-PCR and Western Blot (Figure 1B). For RT-PCR and Western blot, after transcardial perfusion with cold PBS, brains cortex and hippocampus were dissected with ½ of each region processed for western blot and the other half for RT-PCR. A second cohort of female mice (Experiment 2, 27 total) received either water gavage plus vehicle (i.p., *N* = 5), water gavage plus minocycline (Min, 30 mg/kg, i.p., *N* = 5), AIE plus vehicle (*N* = 9), or AIE plus Min (*N* = 8) from P25-P55 (2-days on 2-days off) as in Experiment 1 (Figure 1C). Min was given 30 min prior to ethanol as reported previously (Qin and Crews, 2012). Behavioral testing began on P190, with sacrifice and tissue collection at the end of the behavioral testing (P270) for neurochemical assays. Mice were anesthetized using sodium pentobarbital (100 mg/kg, i.p.) and perfused transcardially with cold 0.1 M phosphate-buffered saline (PBS). Brains were removed and hemispheres separated. One hemisphere was immediately drop-fixed in cold 4% paraformaldehyde for 1 day followed by fixation in a 30% sucrose solution for immunohistochemistry (IHC). The other hemisphere was dissected for the cortex and hippocampus and flash frozen on liquid nitrogen for protein and RNA analyses (Figure 1D). There was an expected increase in average body weights with age into adulthood from P25 to P200, with no significant differences in weight between control and AIE treatment groups either before, during, or after treatment (Figures 1E,F).

### 2.2 RT-PCR

Frozen tissue was homogenized in Trizol (Invitrogen) and RNA isolated by chloroform extraction, followed by reverse transcription as described previously (Qin et al., 2021). SYBR green PCR master mix (Applied Biosystems, Foster City, CA, United States) was used for RT-PCR analysis. The sequences of the primers used are in Supplementary Table S1. Primer sequences were obtained from PrimerBank database (Wang and Seed, 2003; Spandidos et al., 2008; Spandidos et al., 2010) or were designed using the National Library of Medicine Primer-BLAST tool. Only primers with no predicted non-specific targets and single peak melt curves were used. Genes of interest were normalized to the expression of the housekeeping gene 18S. The ΔΔCt method was used to determine relative differences between control and treatment groups and are expressed as the percent difference relative to controls.

### 2.3 Western Blot

Brain tissue from the cerebral cortex was homogenized in lysis buffer (Tris-HCl, pH 7.5, Sucrose, EDTA, EGTA, 1% Triton X-100, protease, and phosphatase inhibitors). 40 mg of protein was loaded into each lane on SDS polyacrylamide gels and was transferred to PVDF membranes. Membranes were washed in TBS and blocked for 1 h at room temperature (Li-Cor Blocking Solution; 92760001) then were incubated overnight at 4°C with the primary antibodies listed in Supplementary Table S2. Membranes were washed in TBS with 0.1% Tween-20 (Sigma-Aldrich, St. Louis, MO, United States) then were incubated in the appropriate conjugated secondary antibody (Rockland H&L Pre-absorbed). Membranes were washed again in TBS and visualized using LiCor Image Studio Lite Ver 5.2. Western Blots were analyzed using Image Studio Lite software and each protein of interest was normalized to housekeeping protein GAPDH. GAPDH expression was not affected by ethanol treatment. The protein of interest was normalized to GAPDH for each sample and the percent change relative to controls calculated for each blot.

### 2.4 Immunohistochemistry

Free-floating sections (3–5 sections per animal) were washed in 0.1 M PBS, quenched in 0.6% H_2_O_2_ for 30 min to inhibit endogenous peroxidases, then incubated at 70°C in citra solution for antigen retrieval. Sections were blocked for 1 h at room temperature in 4% normal serum with 0.1% Triton X-100 to allow membrane permeabilization. Sections were incubated in the relevant primary antibody (Supplementary Table S2) in blocking solution for 18–24 h at 4°C. The following day, sections were washed with PBS, incubated at room temperature for 1 h with a biotinylated secondary antibody (Vector Laboratories, Burlingame, CA, United States), washed briefly in PBS, then incubated for 1 h in avidin-biotin complex (ABC) (Vectastain ABC Kit; Vector Laboratories). To allow for visualization, nickel-enhanced diaminobenzidine was used as a chromogen (Sigma-Aldrich). Negative control for non-specific binding was conducted on separate sections employing the aforementioned procedures omitting the primary antibody. Tissue was mounted on charged glass slides, dehydrated, and covered for imaging. For microscopic analysis and quantification, representative images were taken using a Keyence BZ-X800 all-in-one Microscope. For each region of interest assessed, images were taken on 3–5 identical sections per subject at a representative location. For each brain region assessed by IHC, sections from similar bregma were selected using the mouse brain atlas (Paxinos and Franklin, 2004) to avoid variability in analysis. Bregma used by region were: Entorhinal Cortex and Subiculum (−3.52 to −4.04 mm), Amygdala (−1.94 to −2.14 mm), CA1 (−2.92 to −3.40 mm). The regions of interest were identified, and immunoreactivity (+IR) quantified in each region using ImageJ Analysis Software and reported as +IR pixels/mm^2^. One female control subject was removed for p-tau-181 IHC, due to enlarged ventricles that resulted in atrophy of the hippocampus.

### 2.5 Behavioral Studies

A second cohort of female mice underwent water or adolescent intermittent ethanol (AIE) treatment with 10-weeks behavioral studies for behaviors relevant to AD in the UNC Mouse Behavioral Phenotyping Core, beginning on P190 using the Core’s published methods (Boyer et al., 2018). Experimenters were blinded to the mouse treatment condition. Behavioral studies were conducted during the light cycle in the following order during the mouse age in weeks: weeks 27–29-locomotor activity and exploration in the open field; weeks 28–30-novel object recognition; weeks 29–31- sociability and social memory in a 3-chamber choice test; weeks 30–32-prepulse inhibition of acoustic startle responses; weeks 31–34-acquisition of spatial learning in the water maze; weeks 33–35-reversal learning in the water maze; weeks 35–37-sleep pattern assessment using Piezo sleep monitors. The assays were presented with the most stressful procedures, such as water maze and single-housing for sleep recording, at the end of the battery, to limit carry-over effects of one test to the next test in the regimen.

#### 2.5.1 Open Field Test

Exploratory locomotor activity in a novel environment was assessed in a 1-h trial in an open field arena (41 cm × 41 cm × 30 cm), which was crossed by a grid of photobeams. The arena was placed inside large, sound-attenuating boxes for the open field test. Counts were taken on the number of photobeams broken during the trial, with additional measures of locomotion (distance traveled) and vertical rearing movements (VersaMax, AccuScan Instruments) assessed. Time spent in the center regions (thigmotaxis) was used as an index of anxiety-like behavior.

#### 2.5.2 Novel Object Recognition Test

The NORT test was conducted in an open field arena with novel objects (glass or ceramic saltshakers). On the day prior to the NORT, mice underwent a 10-min acclimation period to the empty open field arena that was placed on a laboratory bench, outside of the sound-attenuating boxes, in a different room from the open field test to retain the novelty aspect of this test. The following day, two identical novel objects were placed into the open field and mice were given 10 min to explore the two objects. One hour later, one familiar object was replaced with a novel object, and mice were given 10 more min to explore the two objects. For each component, the time spent in proximity to each object (nose within 2.5 cm), the number of sniffs directed toward each object, and total distance traveled were measured using the Ethovision automated image tracking system (Noldus). Two subjects were removed due to a failure in the tracking software during testing (one minocycline and one AIE subject).

#### 2.5.3 Three-Chamber Social Choice Test

Mice were evaluated for sociability and social memory using a three-chamber choice task. In this procedure, mice were first given a choice to spend time in the proximity of a stranger mouse or an empty cage. Stranger mice were wild type C57BL/6 mice of the same sex that the tested mice had never been exposed to previously. One hour later, mice were given a choice between the previously investigated mouse, versus a newly introduced stranger.

##### 2.5.3.1 Sociability

For the sociability test, each mouse first explored the empty social test box for a 10-min habituation period. The mouse then was confined to the center chamber, and two cages [one empty and another containing an unfamiliar sex-matched mouse (stranger 1)] were placed in each of the side chambers. The doors for the center chamber were re-opened, and the test mouse was able to freely explore each chamber for 10 min. The time spent and entries into each chamber, as well as the time spent in close proximity to each cage were measured.

##### 2.5.3.2 Social Memory

One hour after the test for sociability, mice were evaluated for social recognition and memory. The original stranger one mouse and a second unfamiliar mouse (stranger 2) were placed into the cages in each side chamber, and the test mouse chose between spending time in the side containing stranger one versus the side containing the newly introduced stranger 2. As before, time spent and entries into each side of the social test box, as well as proximity to each cage were assessed. Two mice were removed from the analysis, as they did not move from the center at all during the 3-chamber social choice test (one minocycline and one AIE).

#### 2.5.4 Prepulse Inhibition of Acoustic Startle Responses

The acoustic startle test was used to measure hearing ability, reactivity to environmental stimuli, and sensorimotor gating. Mice were placed in a small Plexiglas cylinder seated upon a piezoelectric transducer within a larger, sound-attenuating chamber (San Diego Instruments SR-Lab system). The chamber included a ceiling light, fan, and a loudspeaker for the acoustic stimuli (bursts of white noise). The test session consisted of 42 trials, presented after a 5-min habituation period. Seven different types of trials were presented: no-stimulus, trials with the acoustic startle stimulus alone (40 ms; 120 dB), and trials in which a prepulse stimulus (20 ms; either 74, 78, 82, 86, or 90 dB) occurred 100 ms prior to the startle stimulus. Measures were taken of startle magnitude for each trial, defined as the peak response during a 65-msec sampling window beginning with the startle stimulus, and prepulse inhibition, which occurs when a softer sound (the prepulse) reduces startle in response to a subsequent louder noise.

#### 2.5.5 Acquisition of Spatial Learning in the Morris Water Maze With Reversal Learning

Spatial learning and memory retention were assessed using the MWM. The water maze was a large circular pool (122 cm in diameter) filled with water (45 cm deep, 24°C–26°C), in a room with numerous visual cues. Water in the maze was made opaque with nontoxic white poster paint. Mice were first tested for ability to find a visible escape platform. Mice underwent 4 trials per day, across 2–3 days. For each trial, the mouse was placed in the pool at one of four possible locations (randomly ordered), and then given 60 s to find the visible platform. If the mouse found the platform, the trial ended, and the animal was allowed to remain on the platform for 10 s before the next trial began. If the platform was not found within the 60 s period, the mouse was placed on the platform for 10 s, and the next trial started. Following the visible platform phase, mice were further evaluated for spatial learning in the hidden platform test, and reversal learning with the platform moved to the opposite quadrant of the pool. Measures taken included swimming distance, swimming velocity, and latency to find the platform, *via* an automated tracking system (Noldus Ethovision). One-min probe tests with the platform removed were conducted following the acquisition and reversal learning phases, in order to evaluate quadrant selectivity.

#### 2.5.6 Sleep Patterns Across 9 days

Mice were evaluated for sleep cycles across 9 days. Mice were placed into individual PiezoSleep cages before 12:00 noon on the start day. Measures were taken of percent time sleeping and average length of sleep bouts in 12-h light/dark intervals by the automated PiezoSleep system (Signal Solutions, Lexington, KY, United States). During the light phase on day 7, sleep was disrupted for a 3-h period by placing mice into a series of different settings (activity cage with wheel, open field, holeboard, acoustic startle test, and wire-cage enclosure). Sleep rebound was evaluated in the 12-h dark phase on day 7. Sleep was then measured for an additional 2 days, to determine treatment effects on return to regular sleep patterns.

#### 2.5.7 Statistical Analyses

We performed 2-way ANOVAs on all assessments of single molecules that were done in both males and females with Sidak’s post-hoc multiple comparisons test to assess for main effects of AIE and sex. For analysis of proinflammatory genes in both sexes, 2-way ANOVAs were performed for each gene with Sidak’s post-hoc test and correction for repeated testing using the False Discovery Rate (FDR, threshold of *q* = 1% for significance). For analysis of female DAM microglia genes multiple Mann-Whitney t-tests with FDR correction (*q* = 1%) were performed. For all other preplanned orthogonal contrasts of single mediators done in one sex, t-tests were performed. Individual outliers were identified using the Grubb’s test for outliers (GraphPad).

## 3 Results

### 3.1 Adolescent Ethanol Enhances Early Aβ and Tau Pathology in Adult Female 3xTg AD Mice

**FIGURE 2:**
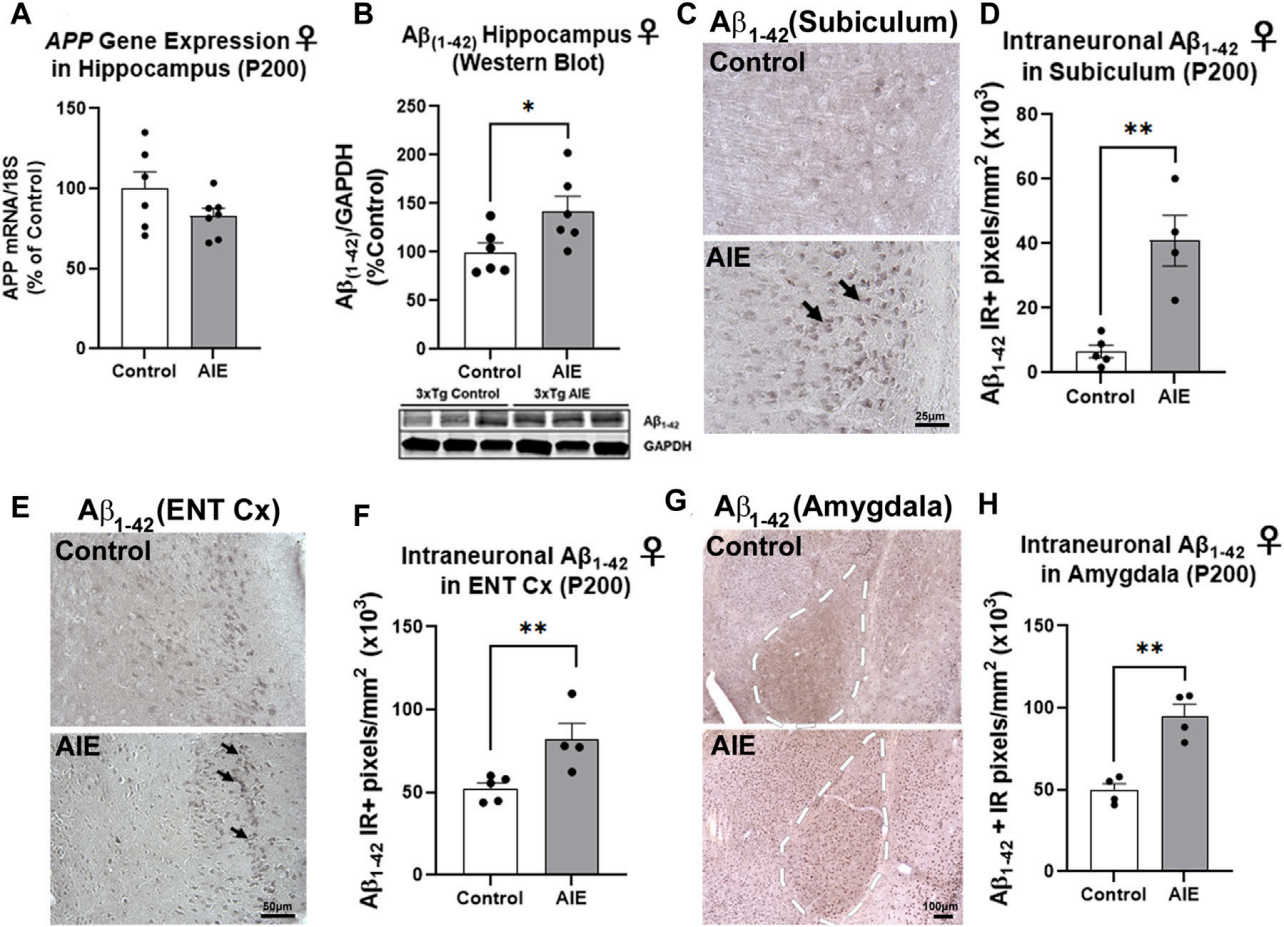
AIE increases amyloid pathology in adult female 3xTg-AD. 3xTg-AD mice received either AIE (5 g/kg/day, i.g., 2-days on 2-days off, P25-55) or water gavage and were assessed for amyloid pathology at P200. **(A)** RT-PCR for human amyloid precursor protein (APP) transgene at P200 found AIE did not significantly alter APP gene expression (*p* = 0.14). *N* = 6 control, 7 ethanol **(B)** Western blot found AIE caused a 42% increase in neurotoxic Aβ_1–42_ protein in female hippocampus (*p < 0.05, Sidak’s post-test). **(C)** Representative image of immunoreactive (+IR) intraneuronal Aβ_1–42_ staining in the subiculum of control and AIE-treated subjects at P200. **(D)** Quantification of intraneuronal Aβ_1–42_ staining in subiculum revealed a 4.8-fold increase after AIE. **p < 0.01, t*-*test. *N* = 5 control, 4 ethanol **(E)** Representative image of intraneuronal Aβ_1–42_ staining in the entorhinal cortex (ENT Cx) **(F)** Quantification of intraneuronal Aβ_1–42_ staining in ENT cortex found a 56% increase after AIE. **p < 0.01 Sidak’s post-test. *N* = 5 control, 4 ethanol **(G)** Representative image of intraneuronal Aβ_1–42_ staining in the amygdala (AMG) of control and AIE-treated subjects at P200, white dashed outline. **(H)** Quantification of intraneuronal Aβ_1–42_ staining in AMG found a 92% (**p < 0.01) increase after AIE. *p < 0.05, **p < 0.01. *N* = 5 control, 4 ethanol.

We first assessed intraneuronal amyloid pathology in these regions to determine if adolescent binge ethanol promotes early AD pathology. Male and female mice underwent AIE with sacrifice at P200, a timepoint characterized by early intraneuronal amyloid accumulation, prior to amyloid plaque deposition. The Aβ_1–42_ cleaved peptide is considered neurotoxic and a precursor to plaque formation (Billings et al., 2005). AIE had no persistent effect on expression of the human APP transgene in the cortex or hippocampus of neither females (Supplementary Figure S1; Figure 2A) nor males (Supplementary Figures S2A,D). In males, AIE did not have any effect on Aβ_1–42_ levels in neither the hippocampus (Supplementary Figure S2B), whole cortex (Supplementary Figure S2C), nor the ENT Cx (Supplementary Figure S2E). Further, there was no sex effect on hippocampal Aβ_1–42_ protein levels by western blot (2-way ANOVA, F_1,21_ = 3.947, *p* = 0.06). However, a significant treatment effect of AIE was found (F_1,21_ = 5.391, *p* = 0.03), with AIE significantly increasing levels of Aβ_1–42_ in female adult hippocampus by 42% (Figure 2B; *p < 0.05, Sidak’s post-test). Western blot analyses of Aβ_1–42_ in whole cortex found no main effects of sex (F_1, 24_ = 1.38, *p =* 0.25) or AIE (F_1, 24_ = 0.63, *p* = 0.43) (Supplementary Figures S1B, 2C). However, IHC found intraneuronal Aβ_1–42_ primarily in the subiculum and entorhinal cortex in neuronal cell bodies (Figures 2C,E), consistent with previous studies (Billings et al., 2005; Gouras et al., 2010). IHC revealed a 4.8-fold increase in intraneuronal Aβ_1–42_ in the subiculum sub-region of the female hippocampus (Figures 2C,D, **p < 0.01, t*-*test). In the ENT Cx, one of the earliest regions impacted by AD, significant main effects of AIE (F_1,12_ = 7.084, *p* = 0.02) and sex were found (F_1,12_ = 15.15, *p* = 0.002) on Aβ_1–42_ immunostaining, with a significant increase due to AIE found only in females (Figures 2E,F, 56% increase, **p < 0.01, Sidak’s post-test). Further, in the basolateral region of the amygdala (AMG), a ∼2-fold increase in intraneuronal Aβ_1–42_ was found (Figures 2G,H, **p < 0.01, t*-*test). Thus, adolescent binge ethanol robustly increases early amyloid pathology in key AD-associated regions in the adult female 3xTg-AD mouse.

**FIGURE 3:**
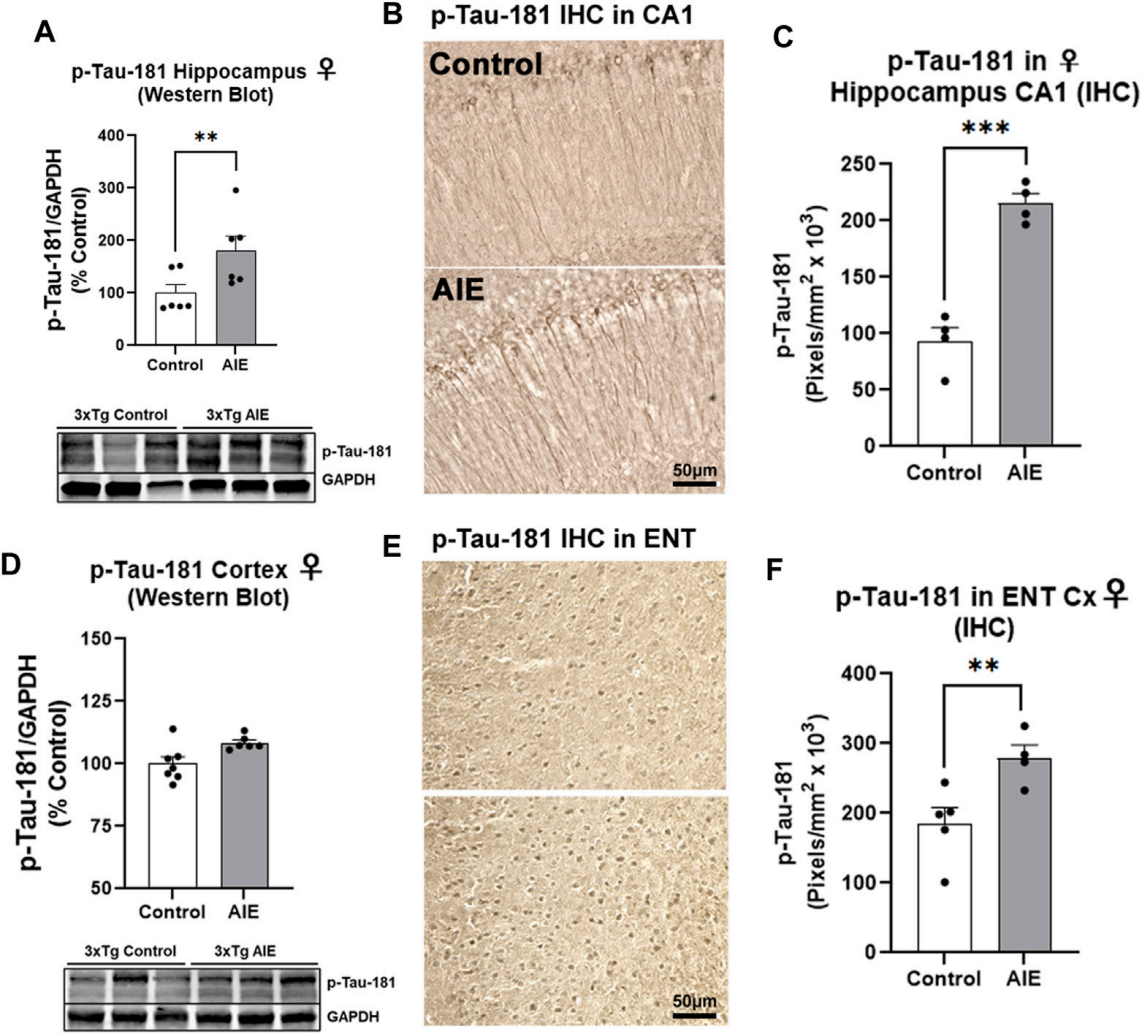
AIE enhances tau pathology in adult female 3xTg-AD mice. 3xTg-AD mice received either AIE (5 g/kg/day, i.g., 2-days on 2-days off, P25-55) or water gavage and were assessed for tau pathology at P200. **(A)** Western blot of adult hippocampus found an 80% increase in phosphorylated tau at Thr181 (p-tau-181) after AIE. **p < 0.01, Sidak’s multiple comparisons test. *N* = 6 control, 6 ethanol **(B)** Representative high magnification image of p-tau-181 staining in CA1 of control and AIE-treated subjects at P200. **(C)** Quantification of p-tau-181 staining in female CA1 found a 2-fold, increase after AIE. **p < 0.01, t*-*test *N* = 4 control, 4 ethanol **(D)** Western blot of adult whole cortex showed no significant change in p-Tau-181 (*p* = 0.21, Sidak’s multiple comparisons test). *N* = 7 control, 6 ethanol. **(E)** Representative high magnification image of p-tau-181 staining in ENT Cx region of control and AIE-treated subjects at P200. **(F)** Quantification of p-tau-181 staining in the ENT Cx found a 51%, (**p < 0.01, Sidak’s post-test *N* = 5 control, 4 ethanol) increase by AIE. *p < 0.05, **p < 0.01.

We next assessed early tau pathology, and measured the tau species that are phosphorylated at the threonine-181 residue (p-tau-181), which is known to be an early biomarker of Alzheimer’s pathology in humans (Wang et al., 2020). Similar to amyloid, AIE had no effect on human tau/MAPT transgene mRNA expression in the male (Supplementary Figure S2F) or female hippocampus (Supplementary Figure S1F). Significant main effects of sex (F_1,22_ = 4.95, *p =* 0.04) and AIE (F_1,22_ = 4.79, *p =* 0.04) on p-tau-181 levels were found in the hippocampus by Western blot, with no significant change seen in males (Supplementary Figure S2G, *p* = 0.98). However, a significant 80% increase in p-tau-181 protein was found in the female hippocampus (Figure 3A, **p < 0.01, Sidak’s post-test). We therefore performed IHC on female subjects and found p-tau-181 + cells were located primarily in the CA1 region of the hippocampus (Figure 3B), with a 2.3-fold increase caused by AIE (Figure 3C, ***p < 0.001, *t-*test). Consistent with findings from the hippocampus, no main effect of sex on whole cortical p-tau-181 was found (F_1,17_ = 0.007, *p =* 0.93) by western blot though a main effect of AIE was detected (F_1,17_ = 5.93, *p =* 0.03) that did not reach statistical significance with post-hoc tests in either sex (Supplementary Figure S2H; Figure 3D). This led us to assess the entorhinal cortex by IHC, the earliest cortical region to be affected by intraneuronal neurofibrillary pathology according to Alzheimer’s Braak Staging (Braak and Braak, 1991), as well as a region highly susceptible to ethanol-induced damage (Tajuddin et al., 2014). Immunohistochemistry revealed a 51% increase in p-tau-181 in the entorhinal cortex in female AIE-treated mice (Figures 3E,F, *p < 0.05, t*-*test). Thus, AIE increases early tau pathology in the female hippocampus and ENT Cx.

### 3.2 Adolescent Binge Ethanol Persistently Increases Proinflammatory Cytokines in Adult Brain That Correlate With Early Aβ and tau Pathology

**FIGURE 4:**
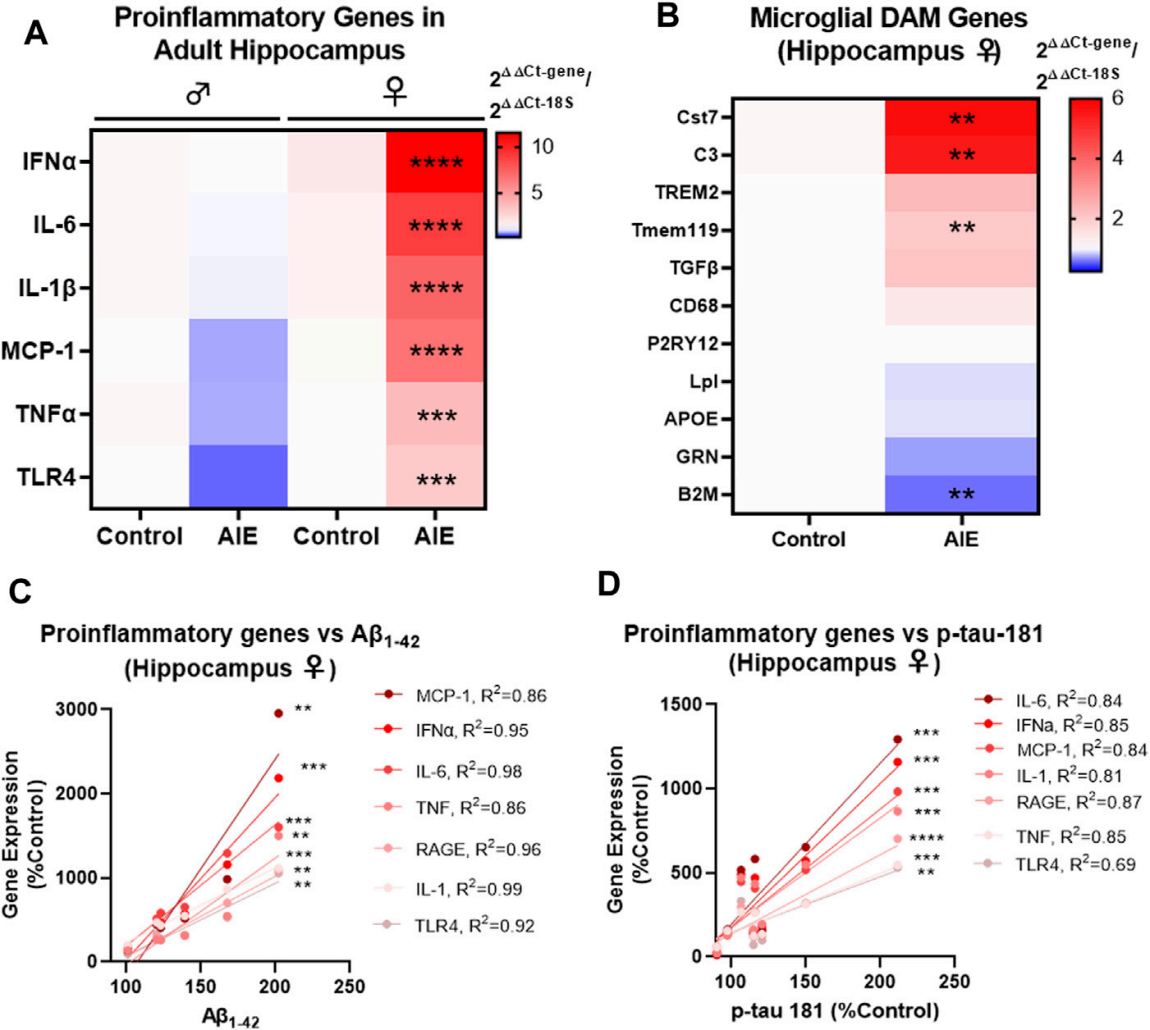
AIE persistently induces inflammation in adult (P200) female brain that correlate with levels of Aβ and tau pathology. 3xTg-AD mice underwent either AIE (5 g/kg/day, i.g., 2-days on 2-days off, P25-55) or water gavage and hippocampus at P200 was assessed for proinflammatory and microglial genes by RT-PCR. **(A)** AIE persistently increased proinflammatory gene expression in IFNα, IL-6, IL-1β, MCP-1, TNFα, and TLR4 in the female hippocampus. 2-way ANOVA with Sidak’s post-test and FDR *p*-value correction, ***q < 0.001, ****q < 0.0001. *N* = 6 control, 7 ethanol. **(B)** AIE significantly altered the expression of microglial DAM genes Cst7, C3, Tmem119, and B2M in the female hippocampus at P200 relative to control. Mann-Whitney *t-*tests with FDR correction, **q < 0.01. *N* = 6 control, 7 ethanol. **(C)** Proinflammatory gene expression was positively correlated with levels of neurotoxic soluble Aβ_1–42_ protein (western blot) in female hippocampus with Pearson R^2^ values between 0.86 and 0.99. **(D)** Proinflammatory gene expression was positively correlated with p-tau-181 protein levels (western blot) in female hippocampus with Pearson R^2^ values between R^2^ = 0.69 and R^2^ = 0.87 with *p* values **< 0.01, ***< 0.001, ****< 0.0001.

Proinflammatory signaling in the brain has been implicated in both AD and adolescent binge ethanol (Eikelenboom et al., 2010; Mathys et al., 2019; Coleman et al., 2021), with recent studies suggesting immune signaling precedes gross AD pathology (Long and Holtzman, 2019). Microglial activation is found early in humans with AD (Streit et al., 2018), with unique disease-associated microglia (DAM) gene sets activated in Alzheimer’s mouse models as the disease progresses (Keren-Shaul et al., 2017). Therefore, we investigated if AIE persistently induces proinflammatory gene and/or alters DAM gene expression in a manner related to AD pathology. Similar to findings above assessing the effects of AIE on AD-pathologic markers, there were significant effects of both AIE and sex on the expression of proinflammatory genes assessed (See Supplementary Tables S3, S4 for complete ANOVA statistical values for each gene). No significant changes were found by post-hoc tests in males in the hippocampus. However, several proinflammatory cytokines implicated in AD were significantly increased in adult female hippocampus by AIE after FDR correction for multiple comparisons including IFNα (11.5-fold, ****q < 0.0001), IL-6 (9-fold, ****q < 0.0001), IL-1β (7.5-fold, ****q < 0.0001), MCP-1 (6.7-fold, ****q < 0.0001), TNFα (3.7-fold, ***q < 0.001), and TLR4 (3-fold, ***q < 0.001, 2-way ANOVAs with Sidak’s post-test and FDR *p*-value correction). A significant effect of AIE was also found on the expression of several DAM genes in the female hippocampus, with significant increases in Cst7 (5.7-fold, **q < 0.01), C3 (5.5-fold, **q < 0.01, Tmem119 (2-fold, **q < 0.01) and a reduction in β2-macroglobulin (B2M, 0.6-fold, **q < 0.01). In the cortex, proinflammatory gene induction was less robust than in the cortex (compare heat map intensities in Figure 4A with Supplementary Figure S1D). However, significant main effects of AIE were found for MCP-1, TNFα, and TLR4 (see Supplementary Table S4 for statistics). Thus, AIE persistently promotes proinflammatory signaling in the female hippocampus while disrupting DAM gene expression. Since inflammatory signaling in the brain is thought to promote AD pathology, we assessed if the expression of proinflammatory genes in the female hippocampus were correlated with early amyloid (Aβ_1–42_ protein by western blot) and tau pathology (p-tau-181, western blot) in that region. Strikingly, levels of the measured proinflammatory genes were strongly correlated with levels of Aβ_1–42_ and p-tau-181 (Figures 4C,D respectively). This included nearly all R^2^ values above 0.8 with several above 0.9. All correlations were significant with all *p* values being at least less than 0.01 and most less than 0.001. This suggested that AIE-induction of proinflammatory genes is related to progression of AD pathology. To determine if AIE-induction of proinflammatory signaling promotes AD progression, we next examined if anti-inflammatory stimulation could prevent AIE-enhancement of AD pathology.

### 3.3 Minocycline Prevents AD-Associated Behavioral Deficits and Neuropathology Caused by Adolescent Binge Ethanol

Given work that suggests proinflammatory signaling contributes to AD pathology (Zhang et al., 2013; Heneka et al., 2015; Jack et al., 2018; Dani et al., 2018; Long and Holtzman, 2019) and our finding that Aβ_1–42_ and p-tau-181 levels were strongly correlated with proinflammatory gene expression after AIE, we hypothesized that blocking proinflammatory signaling during ethanol would prevent AIE-exacerbation of AD pathology. Therefore, we administered minocycline during AIE in an additional cohort of mice. Minocycline is a pharmacological inhibitor of proinflammatory signaling, particularly in glial cells (Attarzadeh-Yazdi et al., 2014; Arezoomandan and Haghparast, 2016; Yew et al., 2019). Since we saw persistent AIE effects on AD pathology and proinflammatory genes in female 3xTg mice but not males, only females were assessed. Mice first underwent behavioral testing in adulthood (P200-270) followed by sacrifice with assessment by IHC (Figures 1C,D). Mice underwent a behavioral testing battery that were chosen to reflect known common symptomatology seen in Alzheimer’s disease such as changes in activity level, anxiety, memory deficits, and social withdrawal (Twamley et al., 2006; McKhann et al., 2011). Thus, the behavioral regimen included baseline activity levels (open field), anxiety-like behavior (thigmotaxis), novel object recognition (NORT), sociability and social memory (3-chamber sociability task), learning and memory retention (Morris Water Maze, MWM), reversal learning, and sleep disruption (Piezo sleep monitoring). Minocycline prevented certain key persistent behavioral deficits caused by AIE in 3xTg-AD mice, implicating immune signaling in the effects of ethanol.

**FIGURE 5:**
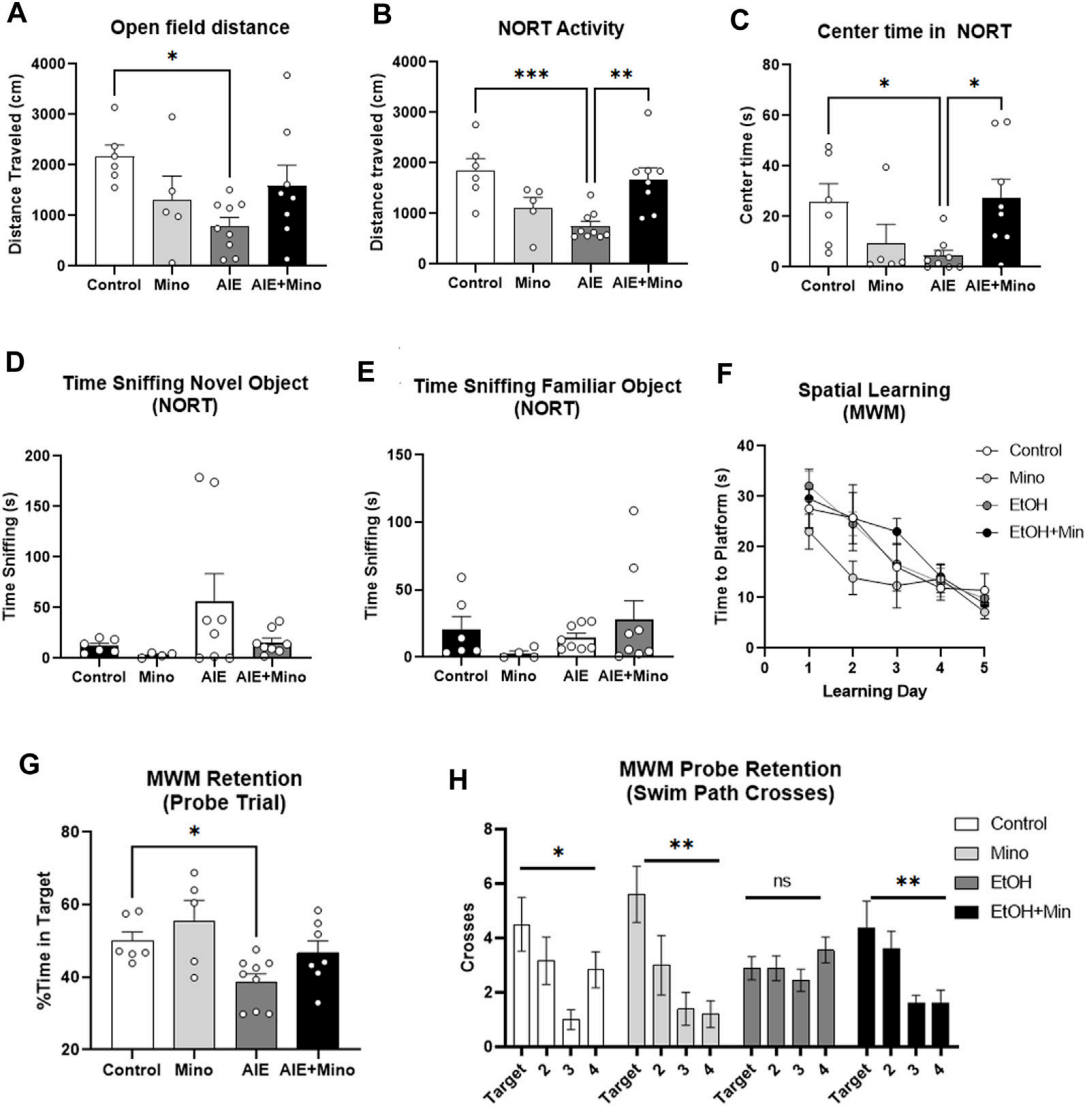
AIE worsens AD-associated activity and memory deficits in adult female 3xTg-AD mice (P200) with prevention by minocycline. **(A, B)** Locomotor activity AIE caused a reduction in locomotor activity in adulthood as measured by **(A)** 1 h in the open field that was blunted by minocycline (63% reduction, 1-way ANOVA treatment effect F_3,24_ = 3.353, *p* = 0.036, *p < 0.05, Sidak’s multiple comparison test), and **(B)** 10 min in the open field during habituation for the novel object recognition test (NORT) that was prevented by minocycline; 60% reduction, F_3,24_ = 7.366, *p =* 0.001, **p < 0.01, ***p < 0.001 Sidak’s post-test. **(C)** Center time in the open field. AIE reduced time spent in the center of an open field with prevention by minocycline as measured by 10 min in the open field during habituation for the NORT; 82% reduction, F_3,24_ = 3.9, *p =* 0.02, *p < 0.01 Sidak’s test. **(D, E)** Novel Object Recognition Test (NORT). **(D)** A trend toward an increase in time sniffing the novel object was seen in the AIE treatment group, *p =* 0.07. **(E)** No significant differences between groups were observed in time sniffing the familiar object during the NORT. **(F, H)** Morris Water Maze (MWM) learning and retention **(F)** AIE had no effect on spatial learning in the MWM. AIE, however, caused an impaired memory retention that was prevented by minocycline as measured during the probe trial with **(G)** less time spent in the correct quadrant; 33% reduction, F_3,23_ = 4.7, *p =* 0.01, *p < 0.05 Sidak’s test, and **(H)** fewer swim path crosses over the target location. 1-way ANOVA treatment effect *p < 0.05, **p < 0.01. *N* = 6 Control, 5 Mino, 8 AIE, 8 AIE + Mino.

AIE reduced baseline locomotor activity in two similar tests – 1 h in the open field and during the 10 min habituation period in the NORT open field. Both tests showed a significant treatment effect on locomotor activity (Figure 5A, ANOVA F_3,24_ = 3.353, *p* = 0.036 and Figure 5B, F_3,24_ = 7.366, *p =* 0.001) with ∼60% reduction caused by AIE (*p < 0.05 in open field and ***p < 0.001 in NORT, Sidak’s post-test) that was prevented by minocycline. In the open field, a trend toward a reduction in time spent in the center by AIE was observed (data not shown, *p =* 0.057). In the NORT habituation period, AIE treated mice showed increased thigmotaxis, spending ∼83% less time in the center as control mice (Figure 5C, F_3,24_ = 3.9, *p =* 0.02), that was prevented by minocycline (*p < 0.05). In the NORT, no differences were found between groups in total time sniffing during training (Supplementary Figure S3A). The AIE group showed a trend toward increased time sniffing the novel object (*p =* 0.07, Figure 5D) with no differences in time sniffing the familiar object (Figure 5E). Regarding learning and memory, there were no significant differences between control and treatment groups in acquisition of spatial learning in the MWM, with all groups finding the escape platform over the 5-day period (Figure 5F). However, mice that received minocycline alone during adolescence did show improved spatial learning during a secondary analysis versus all other groups combined (2-way repeated-measures ANOVA, mixed-effects model, F_1,26_ = 5.227, *p < 0.05). As measured by the post-learning probe trial, mice that underwent AIE had reduced memory retention, spending less time in the target quadrant than control mice (Figure 5G, F_3,23_ = 4.7, *p =* 0.01, 33% reduction, *p < 0.05 Sidak’s multiple comparison test) which was prevented by minocycline. Also, AIE-treated mice showed no signs of increased swim path crosses over the former target platform location (12 cm diameter) versus the corresponding areas in the other quadrants (Figure 5H, F_3,32_ = 1.075, *p =* 0.37). All other treatment groups; however, showed a significant treatment effect on swim path crosses consistent with a non-random search patter and fewer crosses into the corresponding area of the opposite quadrant #3 (control: F_3,20_ = 3.610, *p =* 0.03, minocycline: F_3,16_ = 5.781, *p =* 0.007, and AIE + minocycline: F_3,28_ = 4.832, *p =* 0.008). The AIE + Minocycline group showed a preference for increased path crosses into the former platform target location and the corresponding area in the adjacent quadrant 2 (Figure 5H). Together, measures of time spent in the correct quadrant (Figure 5G) and swim path crosses into the escape target area (Figure 5H) are consistent with protection by minocycline against the deficit in memory retention caused by AIE, though this is not a complete protection. AIE had no effects on reversal learning acquisition (Supplementary Figure S3B), though a slight deficit was seen in the post-reversal learning probe trial in the AIE + mino group (Supplementary Figure S3C, **p < 0.01, t-test). In the 3-chamber sociability test, normal mice typically prefer to investigate a stranger mouse rather than a non-social novel object (i.e., an empty cage) as we have previously reported (Moy et al., 2013). However, in control and Min groups a significant preference for stranger mice was not found (*p =* 0.12 in controls, data not shown), making it impossible to draw conclusions from this test. Further, no differences were seen in prepulse inhibition or sleep patterns (Supplementary Figures S3D,E). Together, these studies indicate that minocycline reduced persistent AIE-effects on locomotor activity, anxiety-like behavior, and memory retention.

**FIGURE 6:**
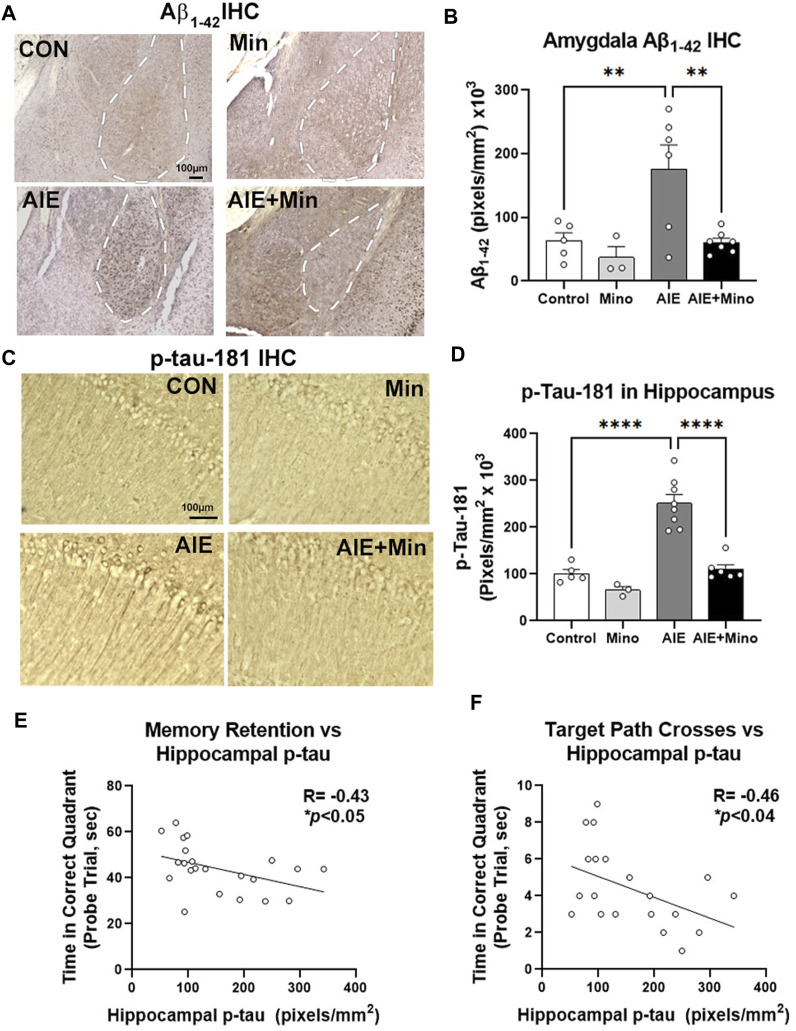
Minocycline reduces AD pathology caused by AIE. 3xTg-AD female mice received either AIE (5 g/kg/day, i.g., 2-days on 2-days off, P25-55) +/− minocycline (30 mg/kg/d) or water gavage. Mice were assessed for amyloid and tau pathology after behavioral testing in adulthood (P270) by IHC. **(A)** Representative images of intraneuronal Aβ_1–42_ staining in the amygdala (AMG, white dashed outline). **(B)** Quantification of intraneuronal Aβ_1–42_ in AMG found a 67% increase by AIE and that was blocked by minocycline. ANOVA F_3,15_ = 15.68, *p <* 0.0001, Sidak’s multiple comparison test. *N* = 5 control, *N* = 3 control + Mino, *N* = 6 AIE, *N* = 7 AIE + mino **(C)** Representative images of p-tau-181 in the CA1 region of hippocampus. AIE increased staining in cell bodies and dendrites of the CA1. **(D)** Quantification of a significant main effect of treatment (F_3,18_ = 31.60, *p <* 0.0001, Sidak’s post-test, *N* = 5 control, *N* = 3 control + Mino, *N* = 8 AIE, *N* = 6 AIE + mino) with a ∼2-fold increase in p-tau-181 by AIE was blocked by minocycline. **(E)** A negative correlation between memory retention and hippocampal p-tau IHC was found. *R* = −0.43, *p < 0.05. **(F)** A negative correlation between target path crosses in the MWM probe trial and hippocampal p-tau-181 immunoreactivity was found. *R* = −0.46, *p < 0.04.*p < 0.05, **p < 0.01 ***p < 0.001, ***p < 0.0001.

Given the relationship between amyloid and tau pathology with behavioral dysfunction in this model, we then measured the effects of minocycline on AIE-enhancement of Aβ and tau by IHC. Similar to our initial findings, a significant treatment effect was found on Aβ_1–42_ in the amygdala (F_3,15_ = 7.03, *p =* 0.0028), with AIE causing a robust 2.8-fold increase (Figures 6A,B, 2.8-fold, **p < 0.01 Sidak’s post-test), and p-tau-181 (F_3,18_ = 31.60, *p* < 0.0001) in the adult hippocampus (Figures 6C,D, 2.5-fold, ****p < 0.0001, Sidak’s post-test) at P270. Strikingly, minocycline administration during AIE blocked these increases, resulting in levels equivalent to age-matched 3xTg controls. Minocycline alone also caused a slight reduction in hippocampal p-tau-181 (35%) and a trend toward a reduction in AMG Aβ_1–42_ (49%, *p* = 0.13). This indicates that binge drinking during adolescence can promote AD-pathology that may be prevented by anti-inflammatory compounds. To assess for age-related changes between the two experiments we compared baseline levels of Aβ_1–42_ in the amygdala and p-Tau-181 in the hippocampus of control subjects. Aβ_1–42_ and p-Tau181 immunoreactivity levels were similar to those observed by IHC at P200 in Experiment 1, with no significant increase in either protein at P270 (Supplementary Figures S4A,B). Since minocycline reversed deficits in memory retention, and blocked increases in Aβ_1–42_ and p-tau-181 caused by AIE, we assessed for correlations between these pathologic protein markers and behavior. Assessments of correlations found that memory retention in the MWM probe trial was negatively correlated with the level of hippocampal tau as measured by time spent in the correct quadrant (Figure 6E, *R* = −0.43, *p < 0.05) and the number of crosses into the target location (Figure 6F, *R* = −0.46, *p < 0.05). This suggests that accumulation of hippocampal tau and amygdala Aβ_1–42_ caused by AIE could promote memory and social deficits associated with AD. Therefore, anti-inflammatory intervention during adolescence with minocycline protects against enhancement of AD molecular and behavioral pathology by adolescent binge ethanol.

## 4 Discussion

Adolescent alcohol abuse is recognized as a risk factor for alcohol use disorder; however, it may also increase risk for cognitive decline with aging (Coleman et al., 2021). Connecting adolescent alcohol use in humans with AD risk can be difficult due to limitations in self-reporting of remote alcohol use. However, recent carefully performed epidemiological studies find heavy alcohol use earlier in life increases risk for AD (Langballe et al., 2015). In the present work, we tested the effects of adolescent binge ethanol on persistent Alzheimer’s molecular and behavioral pathology in a transgenic mouse model that features both amyloid and tau pathology. AIE leads to enhancement of early AD molecular and behavioral pathology in female 3xTg mice. Further, proinflammatory signaling induced by ethanol seems to drive these effects. AIE caused a persistent proinflammatory signature in brain that correlated with pathology, and AIE-induced increases in AD pathology were blocked by the anti-inflammatory compound minocycline. Protection by minocycline was most clearly seen against molecular pathology, as some of the behavioral experiments were underpowered. These findings suggest that induction of proinflammatory microglial signaling during adolescence by alcohol or other insults could be an essential etiological factor in promoting AD pathogenesis.

Recently, paradigms regarding the pathogenesis of AD have begun to be re-examined (Jack et al., 2018; Khachaturian et al., 2018), and multiple studies in humans and rodents implicate neuroimmune signaling in AD pathology (Zhang et al., 2013; Heneka et al., 2015; Dani et al., 2018; Jack et al., 2018; Long and Holtzman, 2019). Proinflammatory insults *in vivo* can also accelerate AD pathology. Previous studies find that LPS-induced neuroinflammation in mice triggers intracellular Aβ accumulation (Sheng et al., 2003; Lee et al., 2008) and tau hyperphosphorylation in the brain (Liu et al., 2016). LPS is a Toll-like receptor 4 (TLR4) agonist that often phenocopies ethanol, which also activates TLR4 (Qin et al., 2013; Lawrimore and Crews, 2017; Crews et al., 2021). Similarly, our findings suggest that persistent proinflammatory activation of microglia by AIE promotes AD pathology in neurons with subsequent behavioral dysfunction. A role for microglial activation in AD has been found in mouse models (Keren-Shaul et al., 2017; Rangaraju et al., 2018), and sustained microglial depletion reduced progression of amyloid pathology in the 5xFAD model (Spangenberg et al., 2019). Several studies find that microglia respond to amyloid accumulation as extracellular plaques develop (von Saucken et al., 2020; Ferretti et al., 2012). However, the mechanisms by which microglia may act on neurons to promote early intraneuronal amyloid pathology are not understood. It is important to note that though the 3xTg-AD model is a transgenic model, and that accumulation of amyloid and tau pathology only occur with aging. This suggests that early in life the animal is able to clear the pathologic proteins, but as the animal ages this capacity is lost. AIE had no effect on the levels of amyloid or tau transgene expression, suggesting that the ability to clear these protein species may be impaired in adulthood. It is also possible that cell death caused during ethanol exposure could contribute to the changes in behavioral function. However, since neuropathological assessments were performed weeks after ethanol treatment, neuronal death would be difficult to assess, which is a limitation. Our results implicate microglia as an initiating factor in promoting early neuronal AD pathology. Previous work from our laboratory and others has implicated microglia as key mediators in response to binge alcohol (Walter and Crews, 2017; Bekhbat et al., 2021). These results suggest that microglia may be persistently “primed” by inflammatory insults earlier in life, which could play a crucial role in the pathogenesis of AD (Li et al., 2018). Microglia can be primed by various stressors or proinflammatory insults, resulting in heightened activation upon subsequent insult (Niraula et al., 2017; Neher and Cunningham, 2019). Since binge alcohol use is more common during adolescence, this may represent a critical period of vulnerability for proinflammatory priming, increasing the risk for neuropathology later in adulthood.

Adolescence is a critical time for neurodevelopment and perhaps neuroimmune maturation. Rodent and human studies show that the transcriptional signatures of glia and expression of several markers associated with inflammation, like Toll-like receptors (TLRs), change across the lifespan (Kaul et al., 2012). TLRs implicated in alcohol abuse such as TLR4 and TLR7 (Montesinos et al., 2016; Qin et al., 2021) undergo a developmental downregulation across adolescence. Further, the number of microglia increases in the rodent brain during adolescence (to a greater degree in females) (Schwarz et al., 2012) and the microglial phagocytic marker CD68 increases across adolescence in human brain (Mildner et al., 2017). AIE persistently increased proinflammatory microglial markers in the female hippocampus. Other environmental insults during adolescence can also promote priming, such as chronic stress, which increases microglia responsive to LPS in adulthood (Bekhbat et al., 2021). However, it remains unclear if glial changes during adolescence create a unique developmental period with heightened vulnerability to immune stimuli than other developmental periods.

It is important to note key limitations of these studies. One limitation of this study is that we only used one ethanol treatment regimen and one dose of minocycline. The dose of minocyline was selected from prior work that found this compound blocks binge ethanol-induced inflammatory gene induction in adults (Qin and Crews, 2012). It is possible that lower doses of minocycline might also be effective. The protection of minocycline against AIE-induced worsening of memory function was not complete protection, and minocycline did not protect against the reversal learning-associated memory deficit, a well-established behavioral finding after adolescent binge ethanol (Coleman et al., 2011; Coleman et al., 2014; Crews et al., 2019). Further, neither AIE nor minocycline had any effect on prepulse inhibition or sleep patterns.

We used the AIE protocol from the NADIA consortium, which models the episodic binge-drinking seen in human adolescents that has been used in many pre-clinical studies (Crews et al., 2019). The effects of other drinking patterns during adolescence on these endpoints are unknown, however insight can be gained from prior studies. A prior study using a daily binge model with i.p. injection of ethanol (2.5 g/kg) across adolescence (P20-P60) in APP/PSE mice found similar increases in protein levels of Aβ42 in the hippocampus at 6 and 12 months of age, with no effects in wild type mice (Ledesma et al., 2021). A study by Hoffman et al. (2019) with voluntary drinking with lower BACs in adult 3xTg-AD mice showed increased staining of p-tau-199/202 in CA1 of the hippocampus and increased Aβ42/40 protein ratios in cortical brain regions 1 month after ethanol with no effects in wild type mice. This suggests that other ethanol exposure paradigms and perhaps lower levels of ethanol during adolescence could also have persistent effects on AD pathology. It is important to note that in our studies wild type mice were not assessed. The majority of wild-type mouse strains do not develop AD pathology, though they do show signs of normal aging. The use of transgenic models with human AD transgenes permits the study of this uniquely human disease in preclinical models. However, the fact that most mouse strains do not develop AD is fundamental limitation in this area of research. Therefore, here we surmise that adolescent binge ethanol enhances AD pathology but cannot conclude that it drives AD pathology. For this reason, future work will employ mouse models that spontaneously develop human-like AD pathology such as the senescence-accelerated mouse (SAM) model (Butterfield and Poon, 2005) to determine causative effects of ethanol.

The current study elucidated sex as an important biological variable in the outcomes that we assessed. Our results found that AIE caused persistent neuroinflammation and increased expression of Alzheimer’s biomarkers in females, but not males. The reason for this dimorphism is not clear. However, in humans, women have a 2-fold greater risk for AD than men (Seshadri et al., 1997), and have faster rates of cognitive decline and brain atrophy after diagnosis with AD (Ferretti et al., 2018). In the 3xTg mouse model, female mice exhibit greater amyloid burden than age-matched males (Billings et al., 2005; Carroll et al., 2010). These studies suggest this dimorphism may be related to sex hormone levels; however, the complete mechanism is not understood. The changes in AD molecular biomarkers after AIE were strongly correlated with increased proinflammatory mediators due to ethanol in females. However, males showed no such persistent proinflammatory gene induction. These sex-dependent differences in inflammatory response to ethanol could underlie the sex differences in AD pathology. As mentioned above, proinflammatory insults can promote AD protein pathology (Lee et al., 2010; Wu et al., 2015). Women, in general, have higher risk for alcohol-related inflammatory conditions such as liver and heart disease (White, 2020). Further, epidemiological studies find that rates of binge-drinking are increasing rapidly in females (Slade et al., 2016; SAMHSA, 2020; White, 2020). Though the reasons underlying these dimorphisms are unclear, the clinical and public health implications could be profound. Future work is required to dissect the impact of binge-drinking in female adolescents on rates of dementia in adulthood, as well as interventions to reduce levels of binge-drinking. It is also possible that promotion of pathology by AIE might also arise in males at later ages, as females show more rapid disease progression in the 3xTg-AD model. Future studies to elucidate mechanisms by which biological sex may contribute to differences in vulnerability to disease and disease progression are necessary, and could help to identify new treatment approaches for AD.

Future studies will also investigate if AIE persistently promotes microglial proinflammatory activation and accumulation of pathologic AD proteins by an epigenetic mechanism. AIE has been found to cause epigenetic changes in multiple brain regions such as the amygdala and basal forebrain to promote persistent neuronal changes in function and gene expression (Pandey et al., 2015; Macht et al., 2020; Vetreno et al., 2020; Crews et al., 2021). Histone chromatin modifications occur in microglia following immune stimuli and can contribute to their proinflammatory priming (Cheray and Joseph, 2018). Thus, studying epigenetic changes in microglia following AIE could identify approaches to normalize microglial activation status. Since this and other work suggest proinflammatory microglia enhance AD progression, normalization of microglial activation status could potentially be beneficial as a preventative or therapeutic approach. In summary, this work is consistent with emerging epidemiological and preclinical studies that find heavy alcohol use during adolescence and young adulthood may represent an important modifiable risk factor for AD.

## Data Availability

The original contributions presented in the study are included in the article/Supplementary Material, further inquiries can be directed to the corresponding author.

## Supplementary Material

Supplementary Figure S1 Effect of AIE on female cortex AD pathology and gene expression. **(A)** RT-PCR for human APP transgene expression in adult female cortex showed no differences between control and AIE-treated 3x-Tg-AD mice (*p* = 0.69, *N* = 7 control, *N* = 7 AIE). **(B)** Western blot (WB) found no effect of AIE on Aβ_1–42_ protein levels in adult female cortex (*p =* 0.72, *N* = 7 control, *N* = 6 AIE). **(C)** RT-PCR for human MAPT transgene expression in adult female cortex showed no differences between control and AIE-treated 3x-Tg-AD mice (*p =* 0.99, *N* = 7 control, *N* = 7 AIE). **(D)** Adult cortex proinflammatory gene expression in control and AIE-treated 3xTg-AD mice. AIE persistently increased proinflammatory gene expression of MCP-1, TNFα, and TLR4 in the female cortex. (2-way ANOVA with Sidak’s post-test and FDR p-value correction, **q* < 0.05, ***q* < 0.01, male: *N* = 6 control, *N* = 7 AIE; female: *N* = 7 control, *N* = 8 AIE). **(E)** Adult female cortex inflammatory gene expression showed no significant change in microglial gene expression due to AIE. Mann-Whitney t*-*tests with FDR correction, *N* = 7 control, *N* = 8 AIE. **(F)** RT-PCR for the human tau transgene (*MAPT*) found no effect of AIE on MAPT gene expression in female hippocampus (*p =* 0.062, *N* = 6 control, *N* = 7 AIE).

Supplementary Figure S2
**AI**E effect on male Alzheimer’s pathology and gene expression. **(A)** RT-PCR for human APP transgene expression in adult male hippocampus (P200) showed no differences between control and AIE-treated 3x-Tg-AD mice (*p* = 0.56). **(B, C)** Western blot (WB) found no effect of AIE on Aβ_1–42_ protein levels in adult **(B)** hippocampus (*p =* 0.31) or **(C)** cortex (*p* = 0.79). **(E)** RT-PCR for human APP transgene expression in adult male cortex (P200) showed no differences between control and AIE-treated 3x-Tg-AD mice (*p* = 0.96). Representative images for Aβ_1–42_ immunohistochemistry (IHC) in male entorhinal cortex (ENT), arrows highlight stained cell bodies. Quantification of IHC for Aβ_1–42_ showed no change in male entorhinal cortex (ENT Cx) (*p* = 0.59, *N* = 3 control, *N* = 4 AIE). **(F)** RT-PCR for human MAPT transgene expression in adult male hippocampus (P200) showed no differences between control and AIE-treated 3x-Tg-AD mice (*p* = 0.27). **(G, H)** WB found no effect of AIE on p-tau-181 levels in adult male **(G)** hippocampus (p = 0.64) or **(H)** cortex (*p =* 0.69). *N* = 6 control, *N* = 8AIE.

Supplementary Figure S3 Effects of AIE on other behavioral tests. **(A)** No differences in time spent sniffing during NORT training were found across groups. **(B)** Reversal learning. AIE had no effect on acquisition of reversal learning in the MWM. **(C)** Reversal learning retention in the probe trial. Crosses over the reversal target region showed trends for a reduction in Mino and AIE groups, that reached statistical significance in the AIE + Mino group. **(D)** Prepulse inhibition. No differences in PPI were seen across groups. **(E)** Piezo sleep testing. No differences in sleep time during dark and light phases were found across groups. *N* = 5 control, *N* = 5 control + Mino, *N* = 9 AIE, *N* = 8 AIE + Mino.

Supplementary Figure S4 Baseline expressions of Aβ_1–42_ and p-Tau-181 by IHC. **(A)** Comparison of control values from IHC assessments of Aβ_1–42_ in the amygdala at postnatal day 200 (P200) and 270 (P270) showed no significant change in baseline levels of Aβ_1–42_ (*p* = 0.40). **(B)** Control immunoreactivity levels from IHC experiments in the CA1 for p-Tau-181 showed no significant changes from P200 to P270 (*p =* 0.14).
