# Supplementary material for: Adolescent Binge Alcohol Enhances Early Alzheimer’s Disease Pathology in Adulthood Through Proinflammatory Neuroimmune Activation
Source: Front Pharmacol. 2022 Apr 26;13:884170. doi: 10.3389/fphar.2022.884170 (PMC9086457; doi:10.3389/fphar.2022.884170)

Full Western Blot Gels

Figure 2 Whole Western Blots

2A – Female Hippocampus Aβ1-42

Gel A

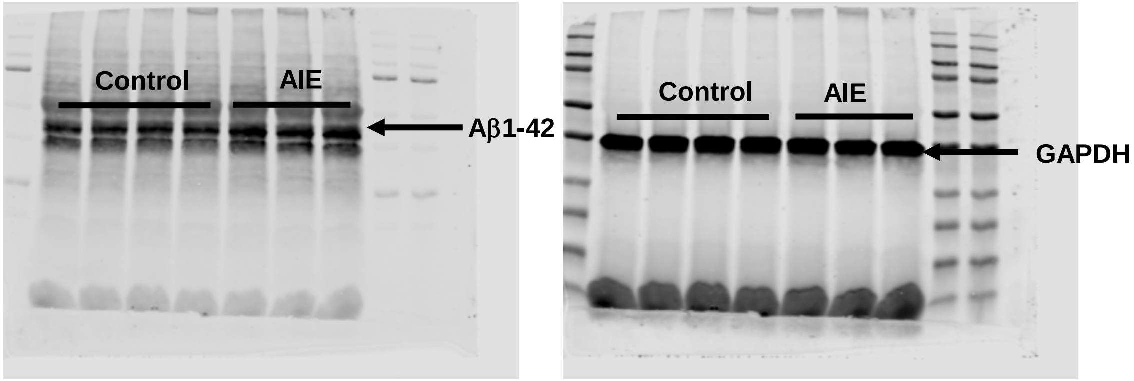

Gel B

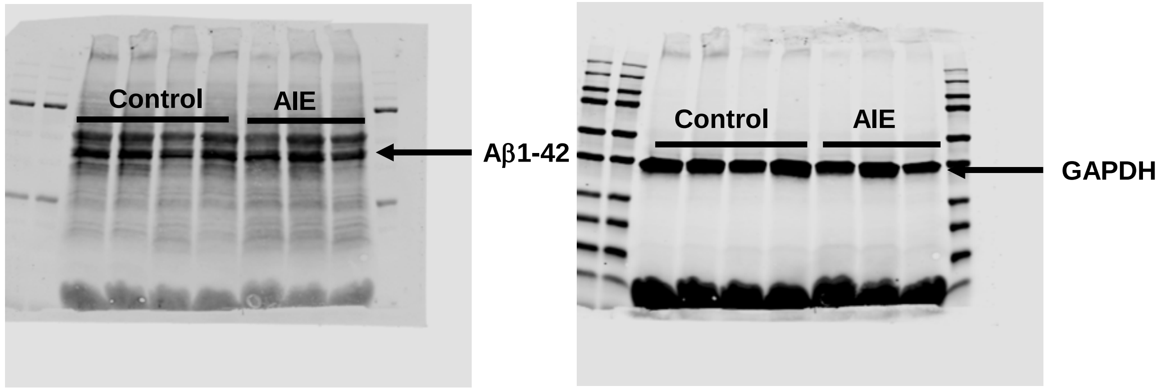

Figure 3 Whole Western Blots

3A – Female Hippocampus p-Tau-181

Gel A

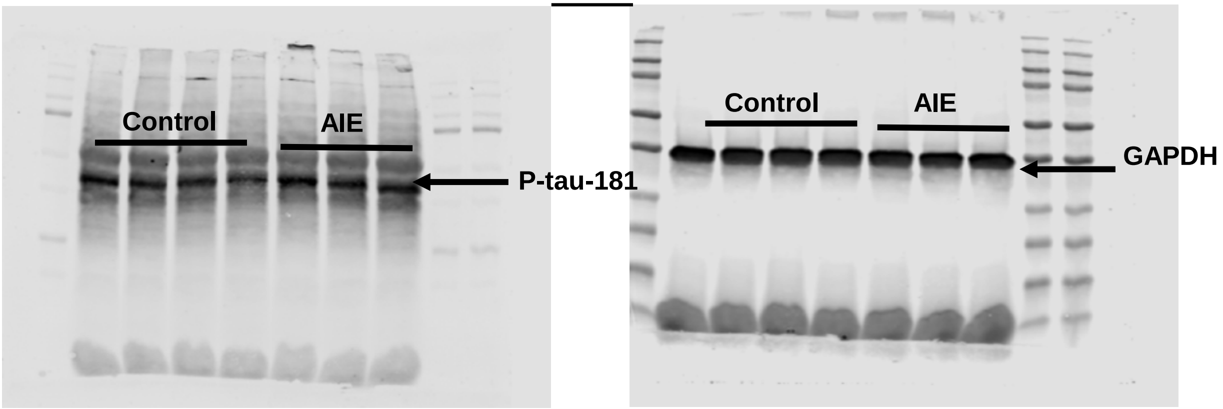

Gel B

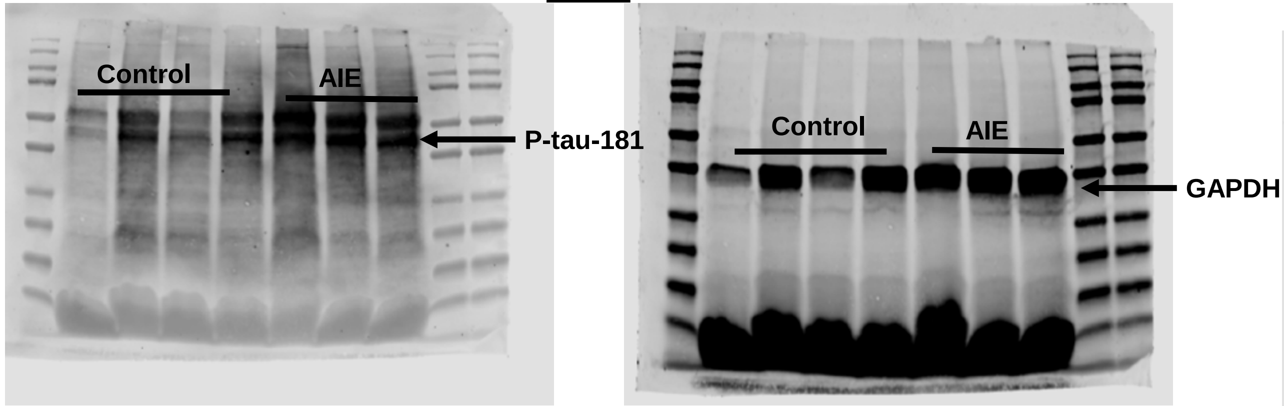

3D – Female Cortex p-Tau-181

Gel A

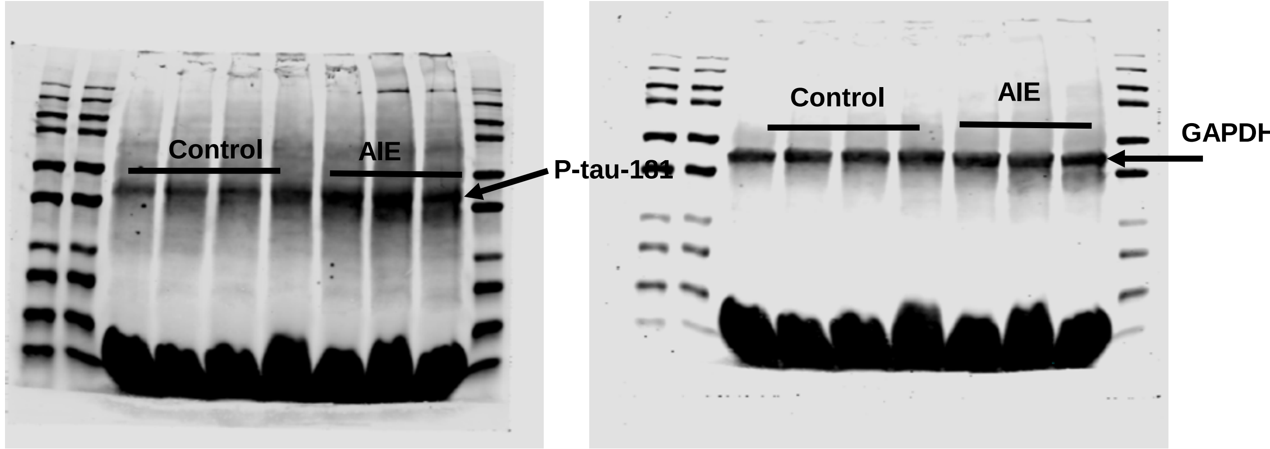

Gel B

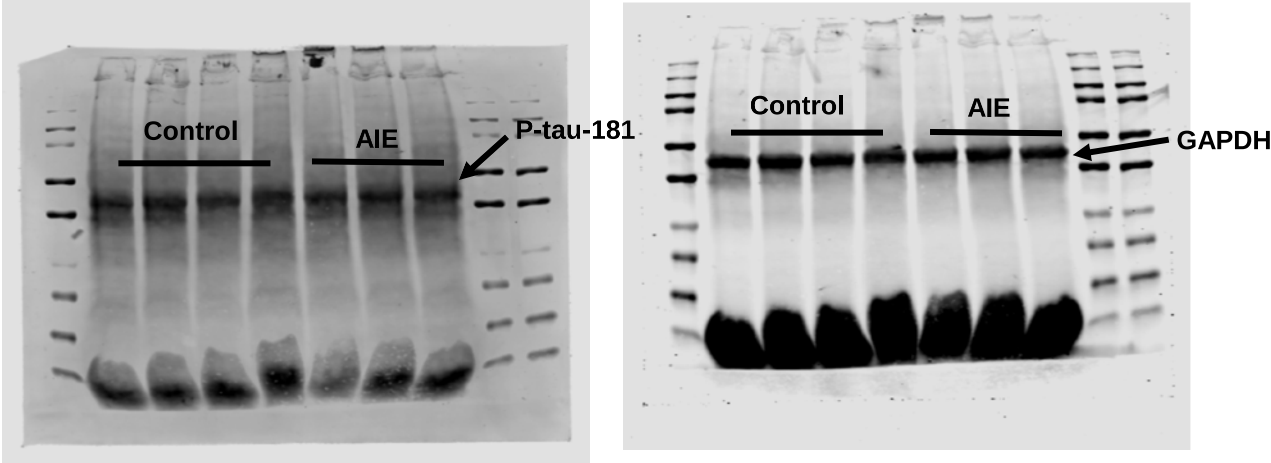

Supplemental Figure 1 Whole Western Blots

1A: Female Cortex Aβ1-42

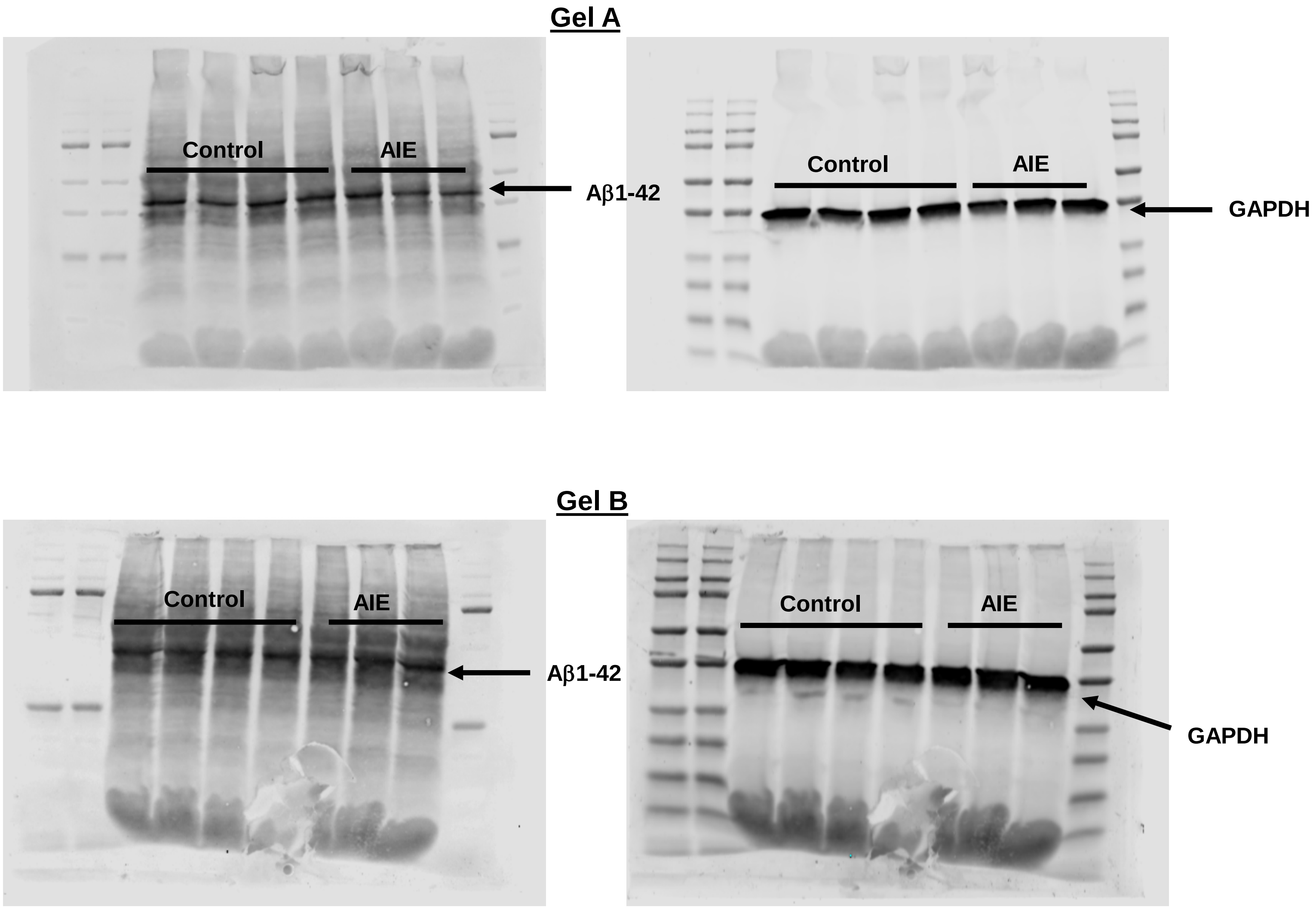

Supplemental Figure 2

2B – Male Hippocampus Aβ1-42

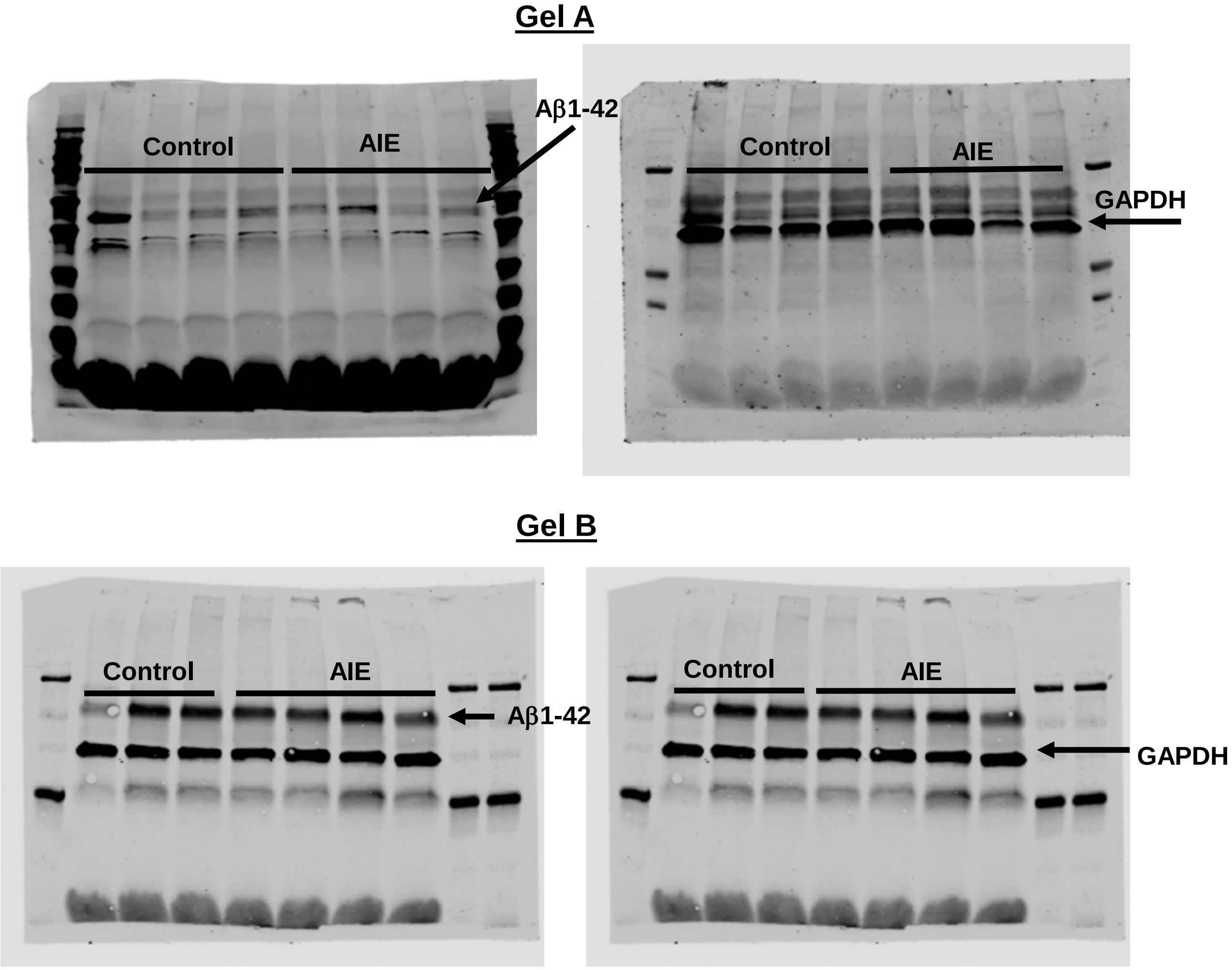

2C – Male Cortex Aβ1-42

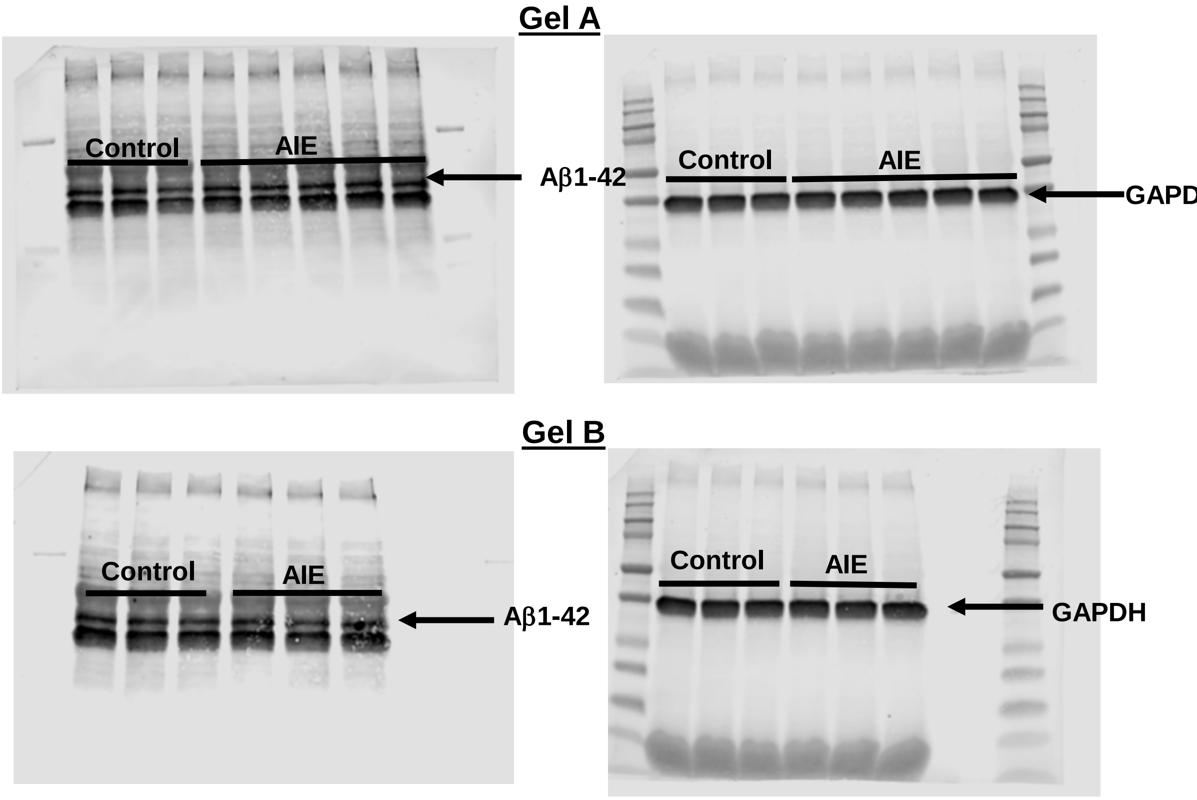

Supplemental Figure 2

2F – Male Hippocampus p-Tau-181

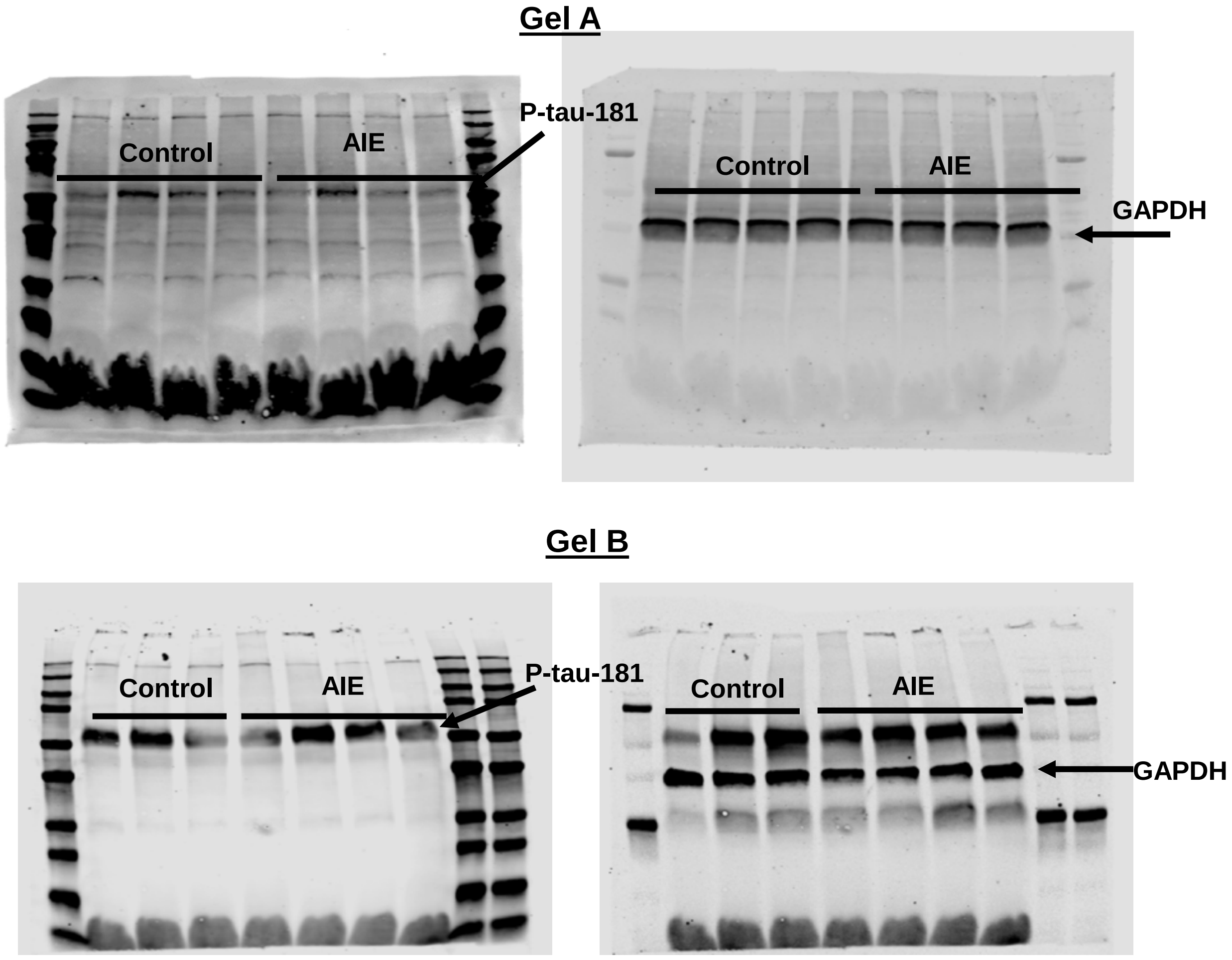

2G – Male Cortex p-Tau-181

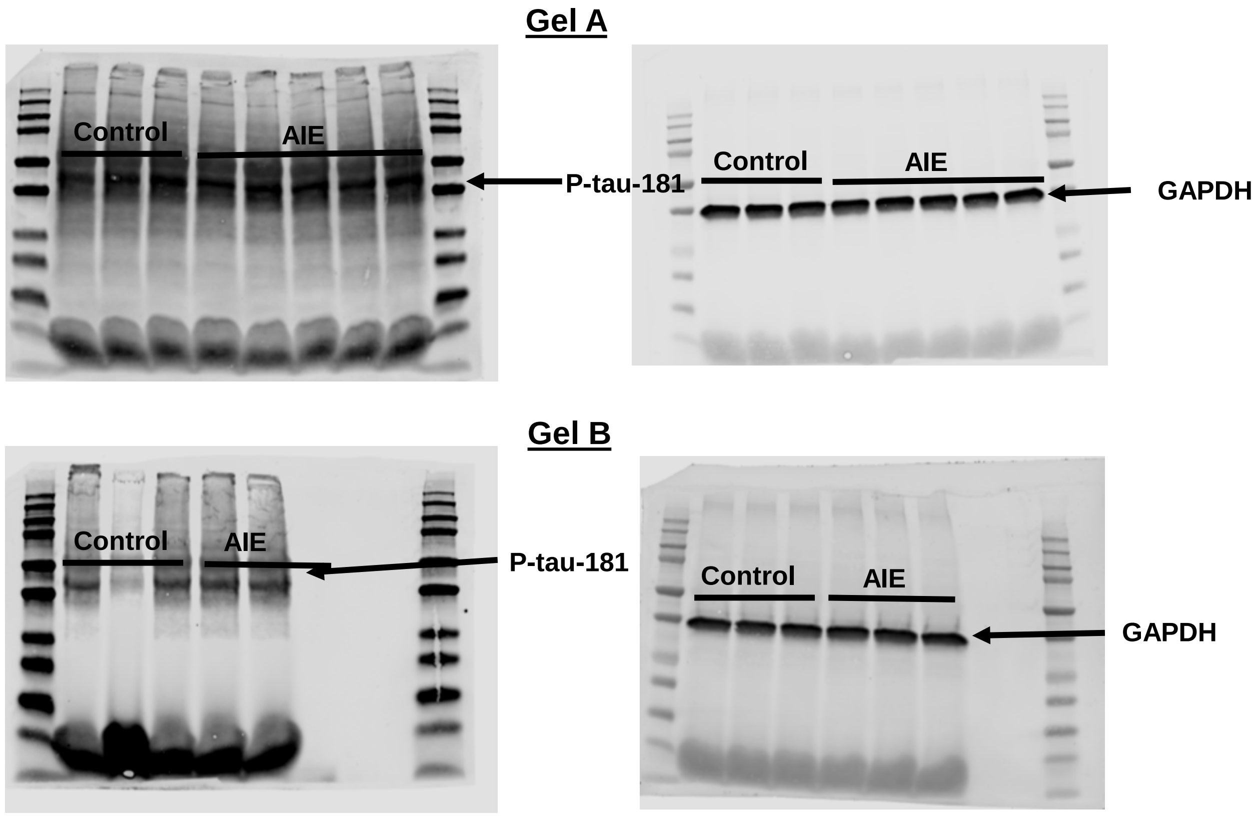

Supplement: Supplementary file 1 [file DataSheet1.zip › Full Western Blot Gels Revised.pdf]
