# Supplementary figures and images for: Adolescent Binge Alcohol Enhances Early Alzheimer’s Disease Pathology in Adulthood Through Proinflammatory Neuroimmune Activation
Source: Front Pharmacol. 2022 Apr 26;13:884170. doi: 10.3389/fphar.2022.884170 (PMC9086457; doi:10.3389/fphar.2022.884170)

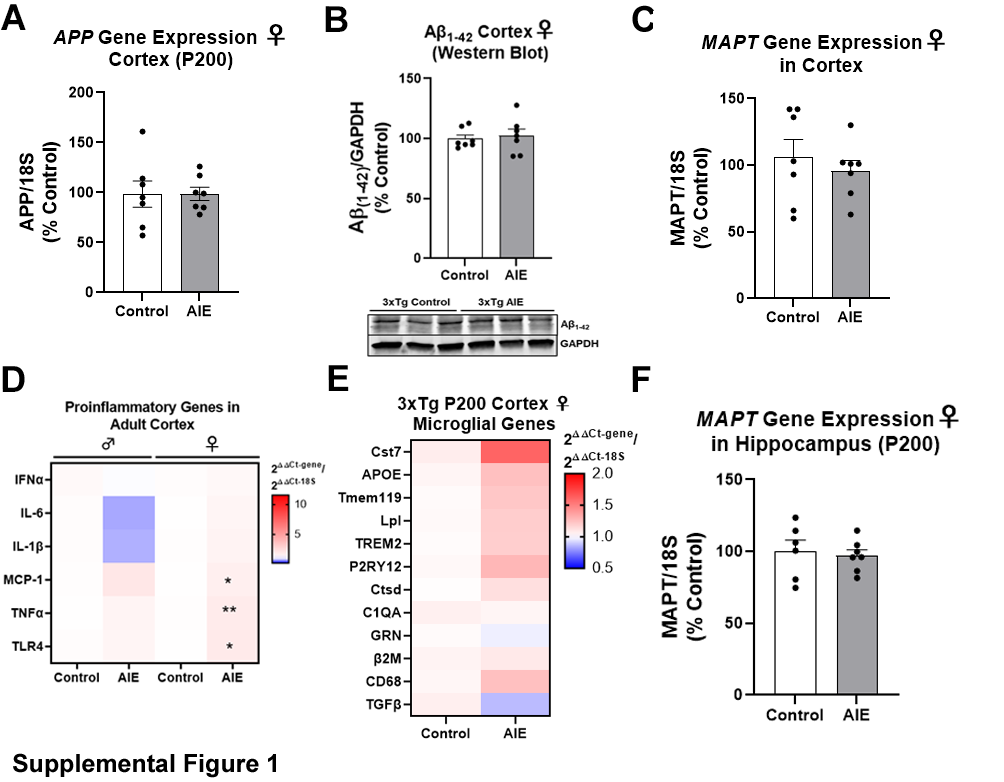

Supplement: Supplementary file 1 [file DataSheet1.zip › Revised Supp Fig 1 - Female Cortex.tif]

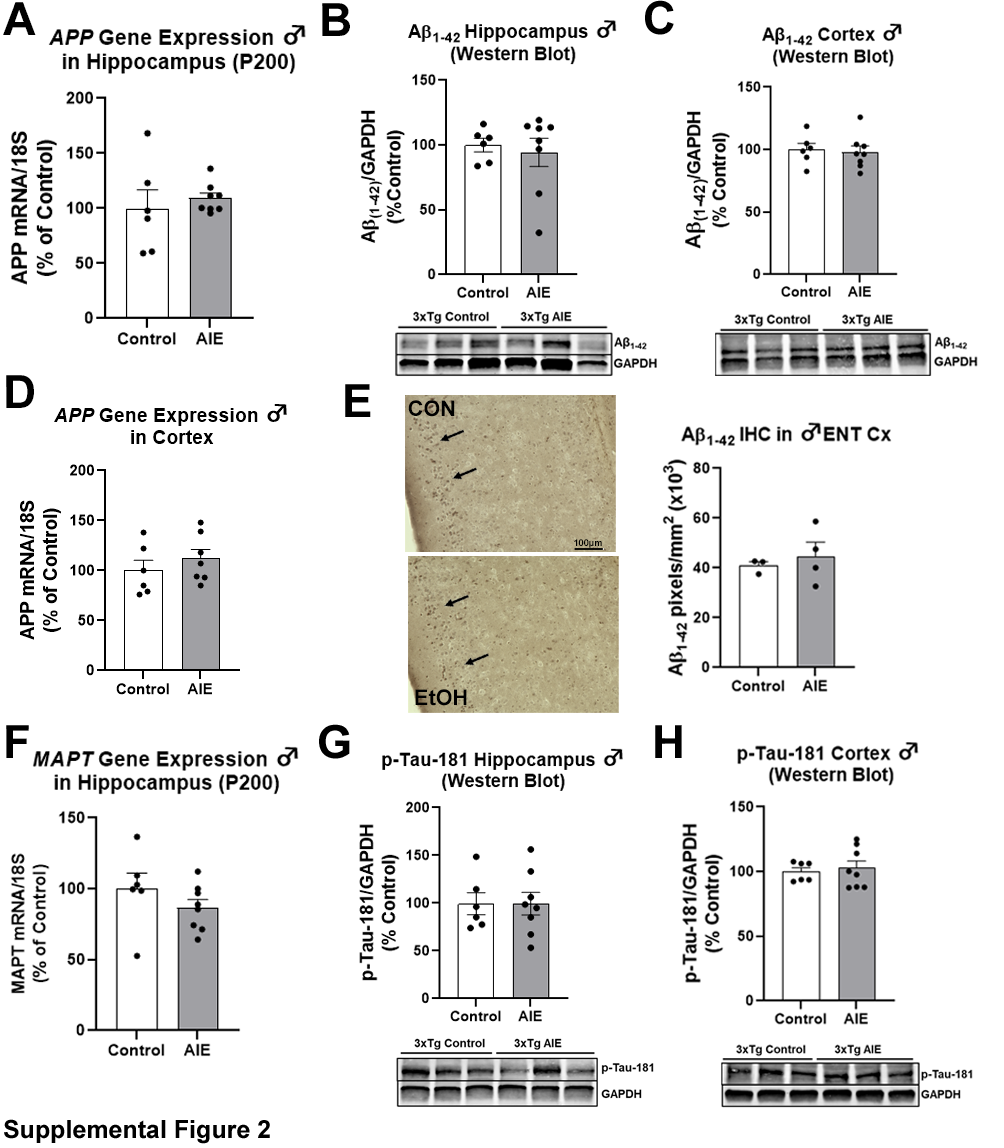

Supplement: Supplementary file 1 [file DataSheet1.zip › Revised Supp Fig 2 - Male Data.tif]

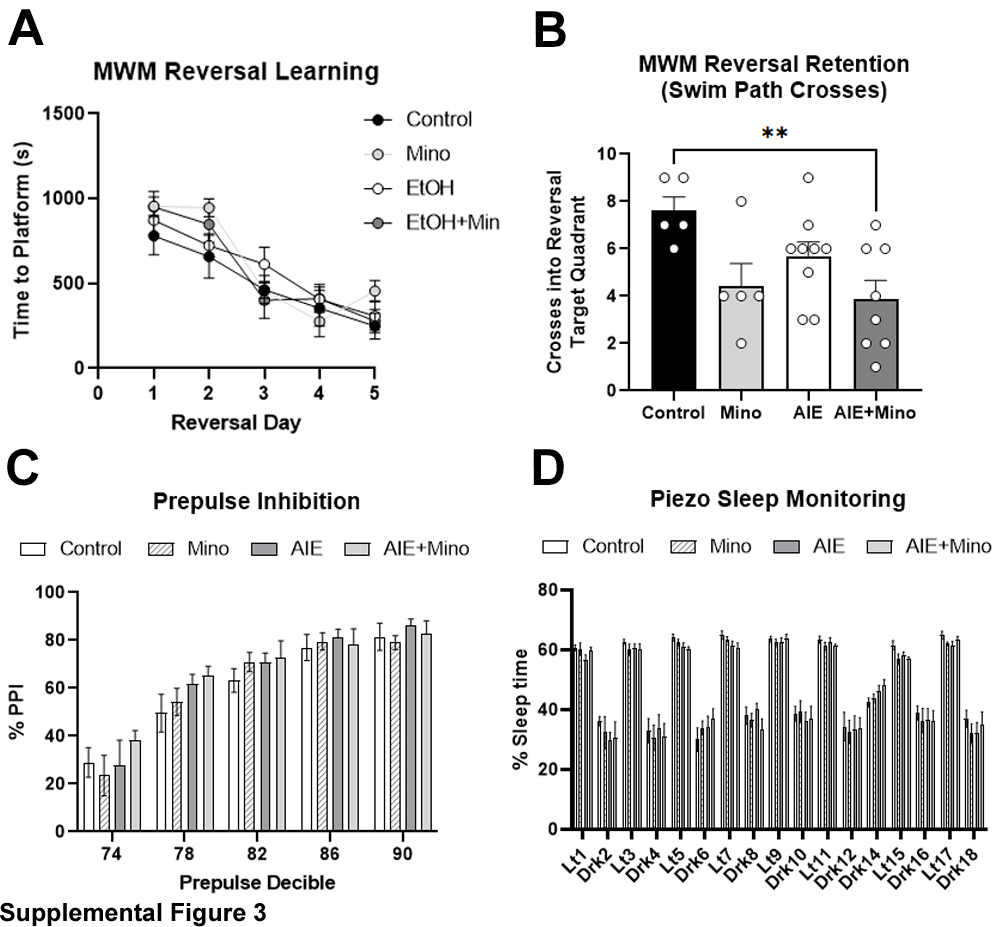

Supplement: Supplementary file 1 [file DataSheet1.zip › Revised Supp Fig 3-Behaviors.tif]

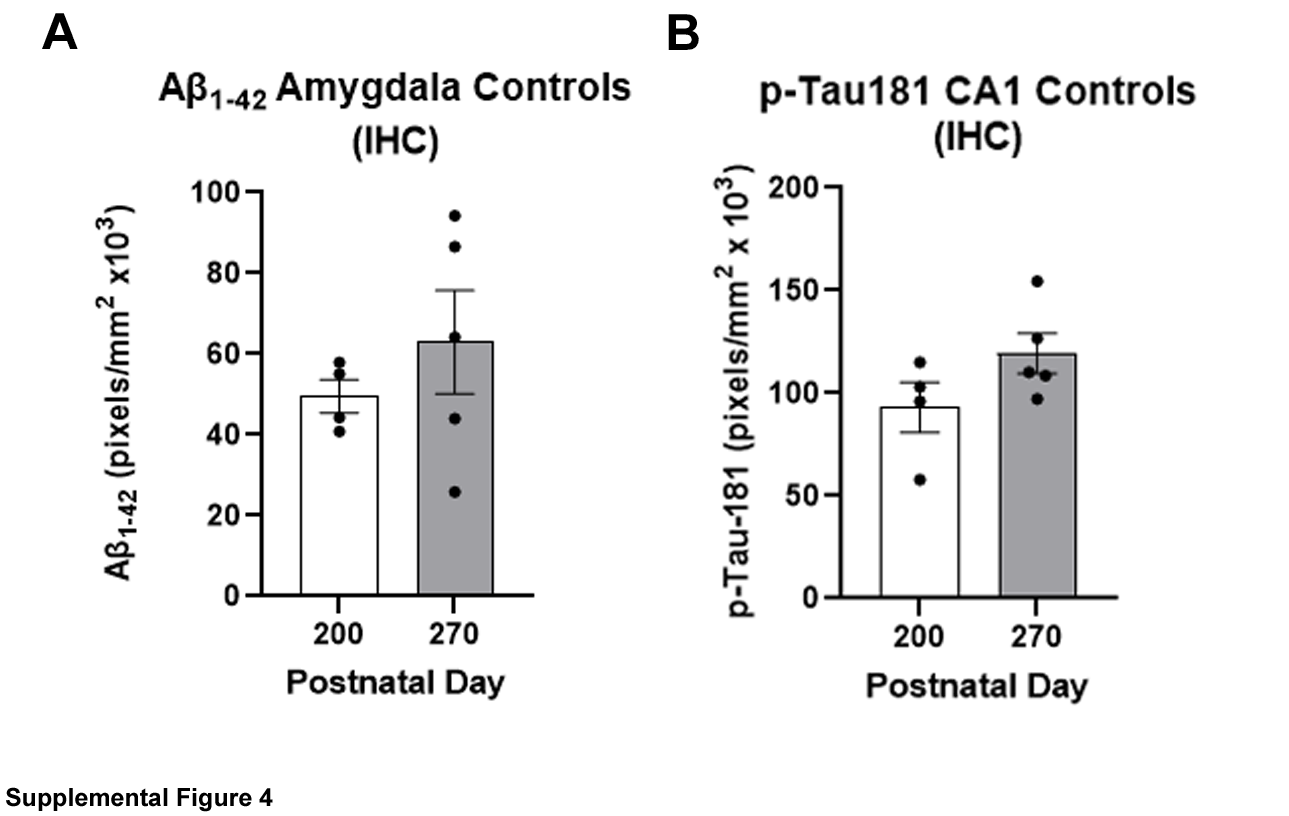

Supplement: Supplementary file 1 [file DataSheet1.zip › Revised Supp Fig 4 - Baseline Control Levels.tif]
